# Using the otolith sulcus to aid in prey identification and improve estimates of prey size in diet studies of a piscivorous predator

**DOI:** 10.1002/ece3.6085

**Published:** 2020-03-23

**Authors:** Barbie L. Byrd, Aleta A. Hohn, Jacob R. Krause

**Affiliations:** ^1^ National Oceanic and Atmospheric Administration (NOAA) National Marine Fisheries Service (NMFS) Southeast Fisheries Science Center Riverside Technology Contractor Beaufort NC USA; ^2^ NOAA NMFS Southeast Fisheries Science Center Beaufort NC USA; ^3^ Department of Applied Ecology Center for Marine Sciences and Technology North Carolina State University Morehead City NC USA

**Keywords:** diet, digestive erosion, estimated prey size, otolith sulcus, prey identification, *Tursiops truncatus*

## Abstract

Diet studies are fundamental for understanding trophic connections in marine ecosystems. In the southeastern US, the common bottlenose dolphin *Tursiops truncatus* is the predominant marine mammal in coastal waters, but its role as a top predator has received little attention. Diet studies of piscivorous predators, like bottlenose dolphins, start with assessing prey otoliths recovered from stomachs or feces, but digestive erosion hampers species identification and underestimates fish weight (FW). To compensate, FW is often estimated from the least affected otoliths and scaled to other otoliths, which also introduces bias. The sulcus, an otolith surface feature, has a species‐specific shape of its ostium and caudal extents, which is within the otolith edge for some species. We explored whether the sulcus could improve species identification and estimation of prey size using a case study of four sciaenid species targeted by fisheries and bottlenose dolphins in North Carolina. Methods were assessed first on otoliths from a reference collection (*n* = 421) and applied to prey otoliths (*n* = 5,308) recovered from 120 stomachs of dead stranded dolphins. We demonstrated in reference‐collection otoliths that cauda to sulcus length (CL:SL) could discriminate between spotted seatrout (*Cynoscion nebulosus*) and weakfish (*Cynoscion regalis*) (classification accuracy = 0.98). This method confirmed for the first time predation of spotted seatrout by bottlenose dolphins in North Carolina. Using predictive models developed from reference‐collection otoliths, we provided evidence that digestion affects otolith length more than sulcus or cauda length, making the latter better predictors. Lastly, we explored scenarios of calculating total consumed biomass across degrees of digestion. A suggested approach was for the least digested otoliths to be scaled to other otoliths iteratively from within the same stomach, month, or season as samples allow. Using the otolith sulcus helped overcome challenges of species identification and fish size estimation, indicating their potential use in other diet studies.

## Graphical abstract

We demonstrated that the otolith sulcus could improve prey identification and limit the bias of digestive erosion in diet studies where correction factors are not available using a case study of four fish species (Family Sciaenidae) targeted by fisheries and common bottlenose dolphins (*Tursiops truncatus*) along the US Atlantic coast.

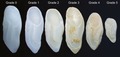

## 1 INTRODUCTION

**Figure 1:**
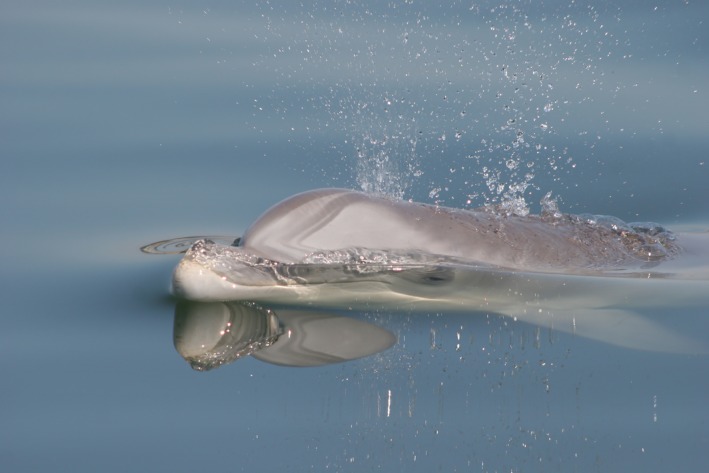
Common bottlenose dolphin (*Tursiops truncatus*) surfacing for air in North Carolina. Photograph taken by staff at the National Marine Fisheries Service, Southeast Fisheries Science Center, Beaufort, NC, under Marine Mammal Protection Act Scientific Research Permit 779‐1633‐00

Understanding trophic connections in marine ecosystems is integral to ecosystem‐based fisheries management (Bowen, 1997; Kenney, Scott, Thompson, & Winn, 1997; Morissette, Kaschner, & Gerber, 2010; Pikitch et al., 2004; Sissenwine & Mace, 2003; Smith, Link, Cadrin, & Palka, 2015; Spitz, Ridoux, Trites, Laran, & Authier, 2018). Piscivorous marine mammals, with their large metabolic energy demands (Williams, Haun, Davis, Fuiman, & Kohin, 2001), are significant consumers of many fish species (Overholtz & Link, 2006; Santos, Saavedra, & Pierce, 2014). Along most of the US Atlantic coast (New Jersey to Florida) and the entire Gulf of Mexico coast, the coastal form of the common bottlenose dolphin *Tursiops truncatus* (hereafter, bottlenose dolphin, or dolphin) (Figure 1) is the predominant marine mammal in estuarine and nearshore coastal waters (Hayes et al., 2018; Hoelzel et al., 1998; Mead & Potter, 1995). Studies on bottlenose dolphin diet indicate that their primary prey are often valuable recreational and commercial fish species (Gannon & Waples, 2004; Pate & McFee, 2012); however, their role as a top predator has received little attention in terms of the effects of their predation on ecosystem dynamics.

For bottlenose dolphins, like many other cetaceans, diet studies often rely on the recovery and identification of hard parts such as fish otoliths (usually the sagitta pair) and cephalopod beaks from stomachs to identify prey and estimate prey size (length and weight) (Fitch & Brownell, 1968; Jobling & Breiby, 1986; Pierce & Boyle, 1991). Otoliths are susceptible to digestive erosion, which can hamper species identification, particularly for closely related species (see Pierce & Boyle, 1991). For bottlenose dolphins along the US Atlantic and Gulf coasts, species in the family Sciaenidae comprise a significant portion of the diet, including at least four species in the genus *Cynoscion* (Barros, 1993; Barros & Odell, 1990; Barros & Wells, 1998; Bowen, 2011; Gannon & Waples, 2004; Pate & McFee, 2012). Otoliths of weakfish (*Cynoscion regalis)* and spotted seatrout (*Cynoscion nebulosus)* look similar. Both species can have crenulated nodules on the distal side of sagittal otoliths (Chao, 1978; Mohsin, 1981), but the nodules have been proposed as more common and pronounced in spotted seatrout than weakfish (Simons, 1986). Nodules may not be a strong predictor of species because of variability in nodules among individuals and age (Simons, 1986). As a result, discriminating between the two species in diet studies often relies on differences in the shape of their sulcus, a surface feature on the proximal side of otoliths that serves as the attachment point of the sensory epithelium (Platt & Popper, 1981). The sulcus in weakfish is slightly longer and narrower compared to spotted seatrout (N. B. Barros pers. comm. ~ June 2009); however, the difference is subtle, making misidentification possible. Misidentification could explain reports of weakfish as dolphin prey outside of its known range (Bowen, 2011; Mercer, 1989) or the lack of reports of spotted seatrout as dolphin prey where both *Cynoscion* species overlap (Gannon & Waples, 2004; Robins & Ray, 1986).

Although the predictive relationship between otolith size and fish size allows for the estimation of original prey size, digestive erosion reduces otolith size resulting in underestimates of fish size, and complete otolith digestion biases the number and species composition of recovered otoliths (Bowen & Iverson, 2013; Harvey, 1989; Pierce & Boyle, 1991). This erosion has cumulative effects on estimates of overall consumption biomass of important prey species for a given predator. As a result, there have been efforts to compensate for the erosion and reduce bias in estimates of prey size. Correction factors have been developed for a variety of prey from many pinniped species in experimental studies (Bowen & Iverson, 2013; Harvey, 1989; Orr & Harvey, 2001; Tollit et al., 1997). The variability in correction factors even for a single predator and prey species limits the application of correction factors across species (Bowen & Iverson, 2013). Additionally, pinniped diet studies typically use hard parts recovered from feces not stomachs, further limiting the applicability of experimentally derived correction factors to cetaceans. Similar experimental studies have not been performed on cetaceans (Bowen & Iverson, 2013). In vitro experiments mimicking digestion in marine mammals have been performed on otoliths to estimate erosion rates (Christiansen, Gamst Moen, Hansen, & Nilssen, 2005; Wijnsma, Pierce, & Santos, 1999), but their application is not straightforward (Andreasen et al., 2017).

In the absence of correction factors, past studies have simply not accounted for digestion erosion (MacLeod, Santos, Lopez, & Pierce, 2006; Santos, Fernandez, López, Martínez, & Pierce, 2007; Santos et al., 2004, 2014; Spitz, Rousseau, & Ridoux, 2006) or have minimized erosion bias by sorting otoliths by the degree of digestion and using the least eroded to estimate fish size (Börjesson, Berggren, & Ganning, 2003; Gannon & Waples, 2004; Recchia & Read, 1989). For the latter, the mean estimated weight of a given prey species from the least eroded otoliths within or across all stomach samples is then applied to the number of otoliths with moderate to severe erosion to estimate overall consumption. This method assumes that original prey sizes are similar between the least and most eroded otoliths. This assumption may not be valid as highly eroded otoliths may consist of originally larger prey whose otoliths require longer to digest (Jobling & Breiby, 1986; Johnstone, Harris, Wanless, & Graves, 1990; Tollit et al., 1997; Wijnsma et al., 1999). Also, otoliths in the same stomach with differing degrees of digestion may represent more than one feeding event (Shippee, 2014) or a single feeding event on schools of differently sized fish (Bowen & Iverson, 2013; DeBlois & Rose, 1996; Tollit et al., 1997; Tollit et al., 2004). Furthermore, the assumption that original prey sizes are similar between the least and most eroded otoliths across samples may not be valid due to variability among individual animals and among sizes for a given prey (Tollit et al., 1997), and to seasonal growth patterns in fish that may influence the size and availability of consumed prey (Axenrot & Hansson, 2004; Claridge, Potter, & Hardisty, 1986). As a result, excluding the more eroded otoliths can introduce bias.

In North Carolina (NC), the sciaenids weakfish, Atlantic croaker *Micropogonias undulatus* (hereafter “croaker”), and spot *Leiostomus xanthurus* were found to be the three most important prey species for the coastal form of bottlenose dolphins (Gannon & Waples, 2004). These species have important commercial and recreational fisheries in the state (NC Division of Marine Fisheries [NCDMF], 2019). Recently, there has been interest from fisheries managers on the drivers and levels of natural mortality due to predation on the weakfish stock along the US Atlantic, which has been decreasing despite decades of harvest restrictions (Atlantic States Marine Fisheries Commission [ASMFC], 2016). To that end, the current study considered ways to ensure accurate discrimination of weakfish and spotted seatrout otoliths recovered from stranded bottlenose dolphins and to improve estimates of prey size.

The sulcus showed promise as a way to aid in species identification and limit the effects of erosion bias. Not only has the sulcus been used to predict fish size (Aguirre, 2003), the shape of its ostium and cauda sections has been used to differentiate species and stocks (Chao, 1978; Torres, Lombarte, & Morales‐Nin, 2000; Tuset, Lombarte, & Assis, 2008). Therefore, we explored whether the sulcus could improve diet studies using a case study of bottlenose dolphins and their prey croaker, spot, and weakfish, and the congener to weakfish, the spotted seatrout. For these species, the perimeter of the sulcus is within the otolith edge perhaps making its length less affected by digestion, thereby reducing bias of estimated prey size (Wijnsma et al., 1999). Using pristine otoliths from a reference collection and otoliths recovered from stomachs of dead stranded bottlenose dolphins, we had four objectives: (a) determine if a quantitative analysis of sulcus shape can distinguish between spotted seatrout and weakfish; (b) develop model equations to predict fish length and weight from otolith, sulcus, and cauda lengths for spotted seatrout, weakfish, croaker, and spot; (c) assess if digestive erosion affects lengths of the otolith, sulcus, and cauda differently; and (d) examine differences in estimated prey consumption biomass for a given prey across three scenarios that exclude or include measurements of otoliths with moderate to severe erosion.

## 2 METHODS

### 2.1 Sample collection and processing

Sagittal otoliths were obtained from two sources: a reference collection (hereafter “reference‐collection otoliths”) and stomachs of bottlenose dolphins (hereafter “prey otoliths”). Reference‐collection otoliths were accessed from a collection of otoliths and squid beaks archived at the National Oceanic and Atmospheric Administration laboratory in Beaufort, NC. The reference collection comprises pristine otoliths removed from fresh or previously frozen fish of species identified using reference guides (Kells & Carpenter, 2011; Robins & Ray, 1986). Reference specimens were collected in a variety of gears (gillnets, hook and line, cast nets, and trawls) across multiple seasons and years primarily in NC estuarine and state coastal waters (<5.6 km from shore). The collection has an accompanying dataset that includes lengths and weights for most specimens. Additional weakfish specimens were loaned by the NCDMF and NC State University (NCSU) to improve sample size. The left and right otolith for each fish were not always available because one side may have broken during extraction or, for samples collected by the NCDMF and NCSU, the left otolith was sectioned and used for aging. Otoliths used for measurements, therefore, were primarily right otoliths unless only the left otolith was available. We assumed measurements between sides did not differ because asymmetry in otolith length between left and right otoliths of round fishes has been demonstrated to be negligible (Lychakov, Rebane, Lombarte, Fuiman, & Takabayashi, 2006; Mille, Mahe, Villanueva, Pontual, & Ernande, 2015).

Prey otoliths were recovered from stomach dissections of 213 dead bottlenose dolphins found stranded in NC between 1996 and 2012. For each dolphin, date of stranding and length measurements was recorded. Date was used to assign dolphins to season: Winter (December–February), Spring (March–May), Summer (June–August), and Fall (September–November). Length was used to assign dolphins to age‐class categories: Young‐of‐year (YOY, <184 cm), older calves (184–211 cm), subadults (212–240 cm), and adults (>240 cm) (Byrd & Hohn, 2017; Fernandez & Hohn, 1998; Read, Wells, Hohn, & Scott, 1993). Extraction of the digestive tract (esophagus to duodenal ampulla) and contents followed established methods (see Gannon & Waples, 2004); however, contents from most strandings were kept separate among digestive compartments (i.e., esophagus, forestomach, main stomach, pyloric stomach, duodenal ampulla). We restricted this study to contents removed from the forestomach and esophagus (Recchia & Read, 1989) because contents from the esophagus are likely regurgitated from the forestomach during death (Harrison, Johnson, & Young, 1970; Pierce & Boyle, 1991), and contents from the forestomach represent the most recent feeding event(s). Also, the continuation of digestion in the main and pyloric stomachs would disproportionately affect otoliths compared to squid beaks and increase the chances that small otoliths would be completely digested (Bowen & Iverson, 2013). This study was intended to include only the coastal form or “morphotype” of bottlenose dolphins, which is genetically distinct from (Hoelzel et al., 1998, Rosel, Hansen, & Hohn, 2009) and has different diet than (Mead & Potter, 1995) the offshore morphotype. As such, samples were excluded from bottlenose dolphins genetically determined to be the offshore morphotype (see Byrd et al., 2014) or thought to be offshore morphotype by the presence of parasites (e.g., *Monorhygma* and *Phyllobothrium*) not found in the coastal morphotype (Mead & Potter, 1995).

**Figure 2:**
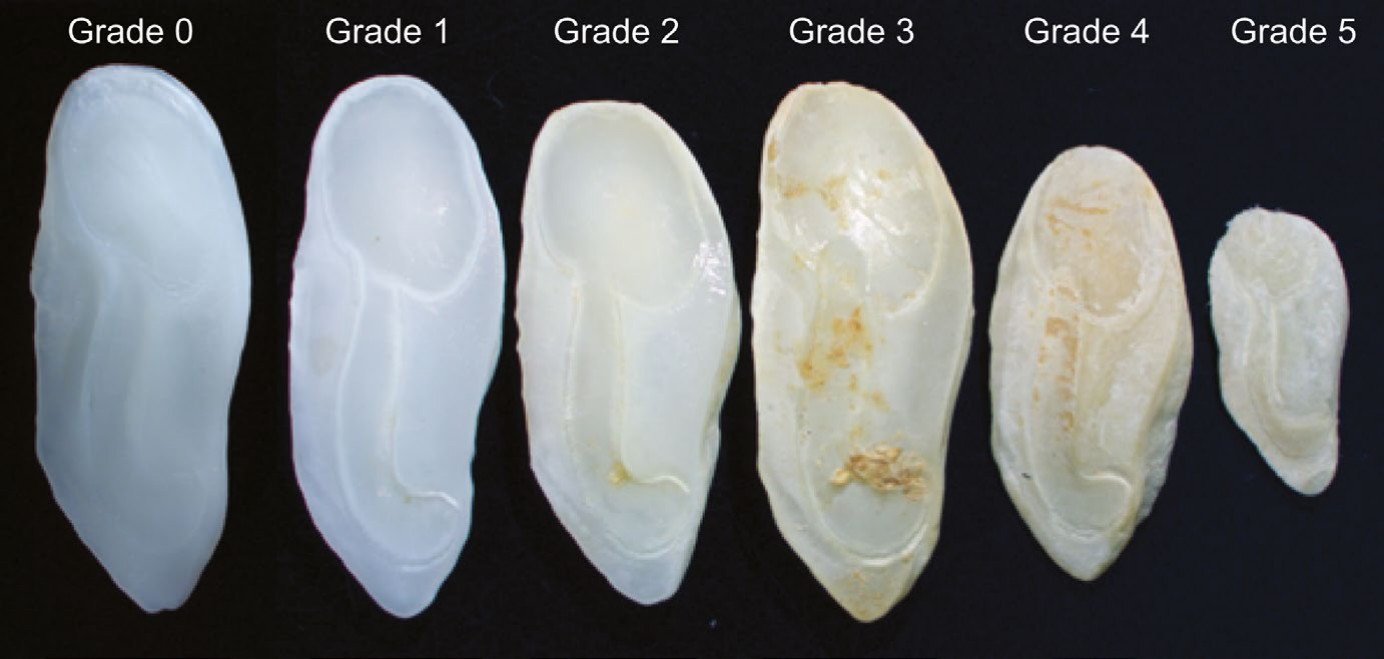
Weakfish (*Cynoscion regalis*) otoliths from the reference collection (Grade 0) and from dolphin stomachs showing grades of digestive erosion (see Table 1 for grade description)

Prey otoliths were identified by comparison to the reference collection and published otolith guides (Baremore & Bethea, 2010; Campana, 2004). To enumerate prey for each dolphin, the left and right otolith of each prey species was counted. The side with the highest count was used to estimate the number of prey consumed for that species (Orr & Harvey, 2001). Afterward, otoliths from that side were graded according to digestive erosion from grade 0 to 5 (adapted from Recchia & Read, 1989; Tollit et al., 1997). Otoliths with no to slight erosion were graded separately: 0, undamaged; 1, barely degraded, sulcus fully visible; and 2, slightly degraded, somewhat chalky appearance, sulcus fully visible (Figure 2). Otoliths with moderate to severe erosion were combined into a single category, Grade 3–5, with no attempt to individually assign grade number. These otoliths ranged from moderate erosion where intricacies of otolith shape showed obvious wear and distance between otolith and sulcus edges was decreased, to severe erosion where the sulcus extent was obviously compromised or the otolith was broken (Figure 2). There were indications that digestive fluids may have entered into fish skulls, which were often found separated from the bodies and in various stages of digestion. As such, we graded all otoliths removed from fish skulls rather than automatically scoring them as grade 0 (undamaged) as has been reported in past studies (Gannon & Waples, 2004; Recchia & Read, 1989). All otoliths graded individually as 0–2 then were combined into one category (Grade 0–2) for analyses due to low sample sizes for grade 0 and grade 1 otoliths.

**Figure 3:**
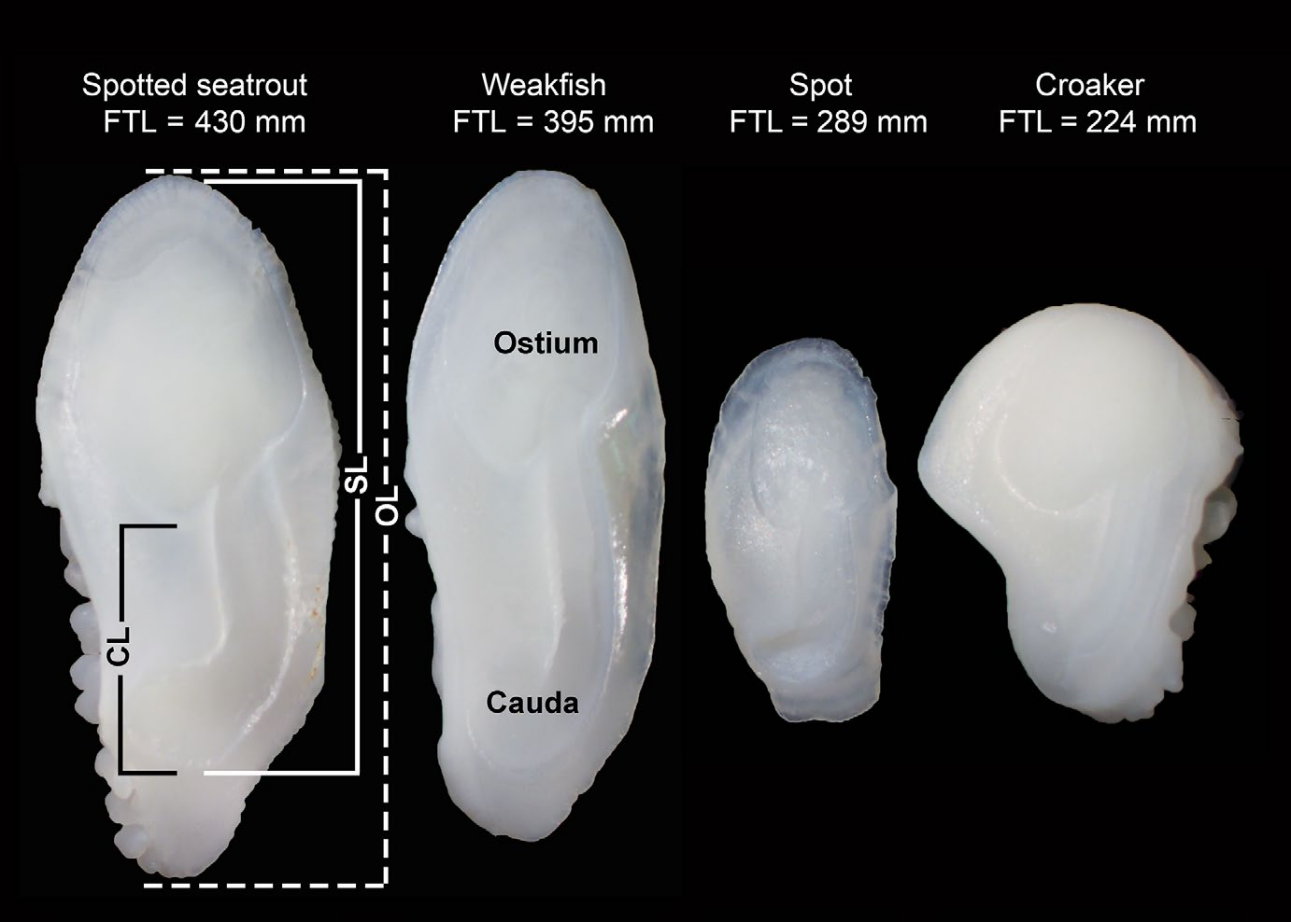
Right otoliths from prey species showing measurements of otolith length (OL), sulcus length (SL), and cauda length (CL). FTL = fish total length. Species are spotted seatrout (*Cynoscion nebulosus*), weakfish (*Cynoscion regalis*), spot (*Leiostomus xanthurus*), and croaker (*Micropogonias undulatus*)

Reference‐collection otoliths and prey otoliths from croaker, spot, spotted seatrout, and weakfish (Figure 3) were measured to the nearest 0.01 mm using a dissecting microscope (Olympus BZ61) at 6.7–10x magnification and imaging software (Olympus cellSens). We took three steps to reduce measurement error. First, the imaging software was set to collect images at the highest resolution (2,448 × 1,920 pixels). The “helper lines” tool was also used for measurements whereby perpendicular lines are shown at the beginning and end of the measured line to ensure correct placement and longitudinal alignment. Lastly, the same person measured all otoliths. Measurements included length of the otolith (OL) and the features sulcus (SL) and cauda (CL) (Figure 3). For these species, CL was chosen as a predictor over ostium length because the cauda was further from the otolith margin. The CL was measured between the inferior point of the ostium and inferior point of the cauda. Because of the large number of prey otoliths graded 3–5, a subset was measured for croaker (*n* = 120) and spot (*n* = 101). In case prey size is influenced by the size (thus, age) of the dolphin predator, the subset was first chosen by proportional representation across dolphin age‐classes and the unknown age‐class for dolphins without total lengths. Within each age‐class category, dolphins and measured otoliths were chosen at random to best represent the sizes contained therein. Any otolith obviously compromised (e.g., broken or sulcus extent missing) was not measured. To assess the direction of erosion bias on SL and CL, otoliths from each species were examined by eye and using the dissecting microscope to assess the angle of sulcus walls (i.e., outward angle would overestimate prey size, whereas inward angle would underestimate prey size with erosion). In each case, the otoliths were examined across the longitudinal plane of the otolith.

### 2.2 Discrimination between spotted seatrout and weakfish

**Figure 4:**
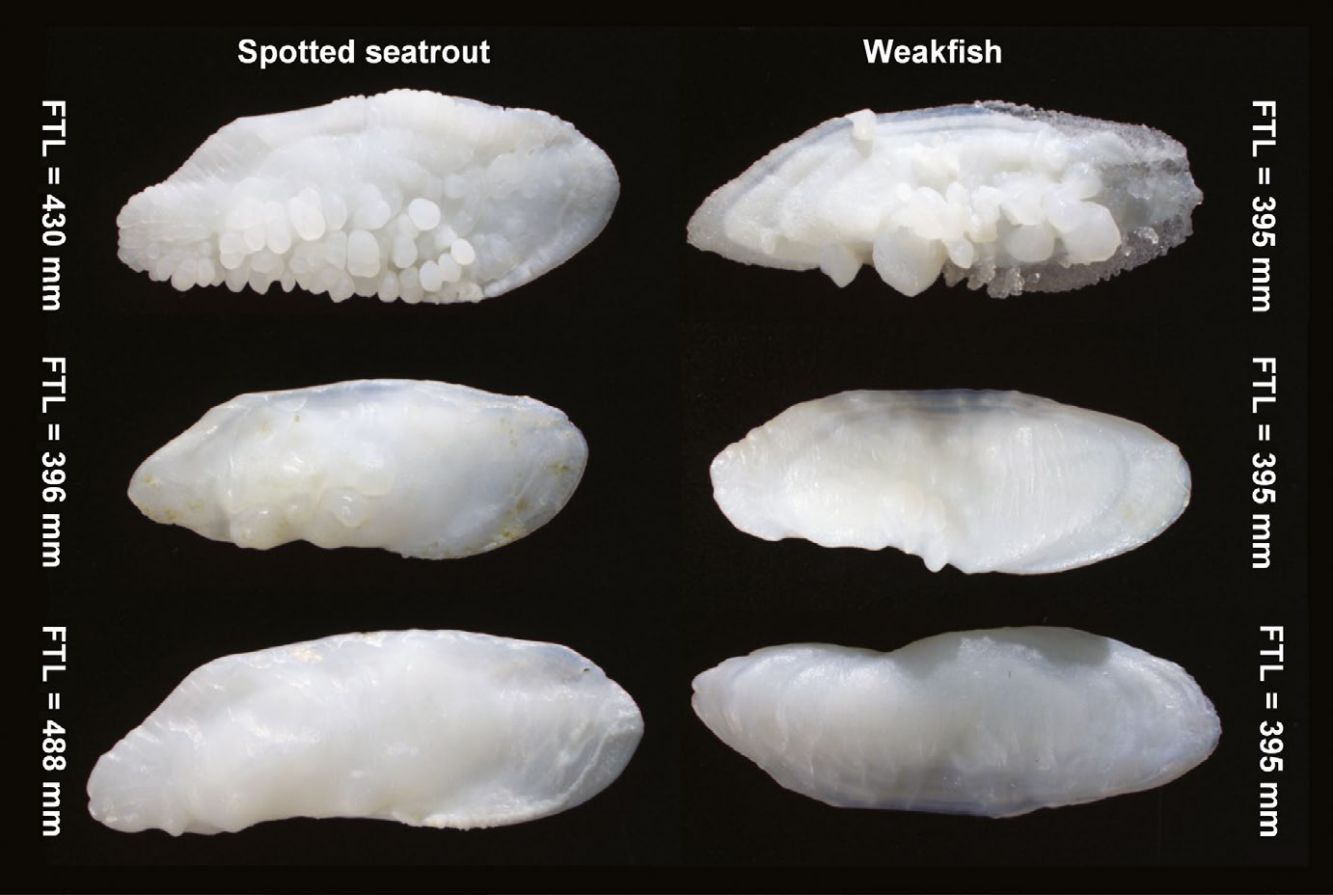
Spotted seatrout (*Cynoscion nebulosus*) and weakfish (*Cynoscion regalis*) otoliths showing prominence of nodules: present (top), reduced (middle), and absent (bottom). For weakfish, the top and bottom otoliths are from the same fish. FTL, fish total length

**Figure 5:**
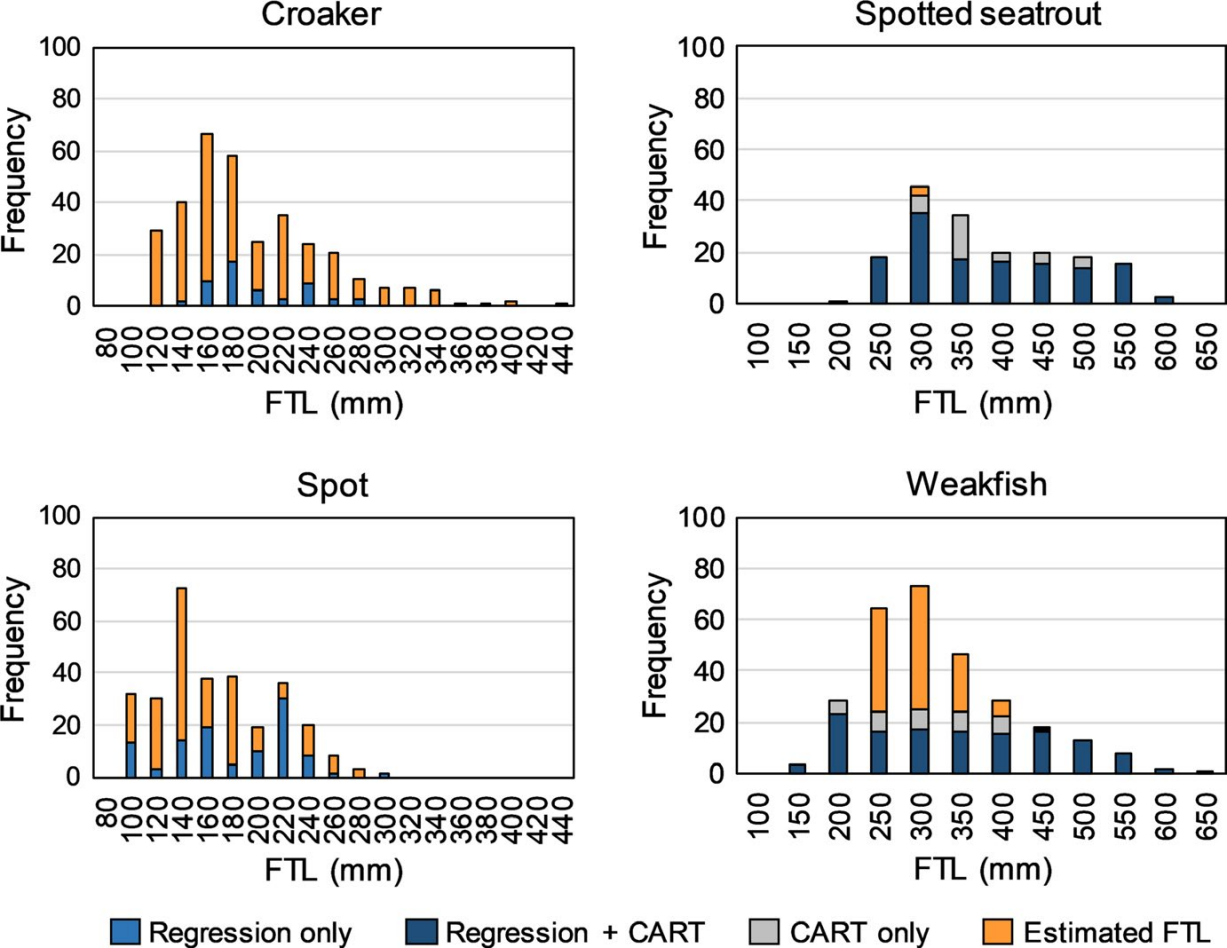
Distribution of fish total length (FTL) measured from reference‐collection fish samples and estimated from prey otoliths recovered from dolphin stomachs. Otoliths measured from reference‐collection fish were used in regressions to predict fish size from otolith length (OL), sulcus length (SL), and caudal length (CL), for the shape analysis (Classification and Regression Tree [CART]), or both. For prey otoliths, estimated FTL was from measurement of otoliths Grade 0–2 using prediction equations from reference‐collection otoliths. Sulcus length was used to estimate FTL of croaker (*Micropogonias undulatus*); cauda length was used to estimate FTL of spot (*Leiostomus xanthurus*), spotted seatrout (*Cynoscion nebulosus*), and weakfish (*Cynoscion regalis*). Note x‐axes scaled differently

We assessed two otolith characteristics on reference‐collection otoliths to discriminate between spotted seatrout and weakfish prey otoliths: sulcus shape and nodules. For both sample types (reference collection and prey otoliths), we quantified the difference in sulcus shape between the two species by using the ratio CL:SL. We qualitatively assessed the presence and relative prominence of nodules by categorizing reference collection and prey otoliths as having nodules present, nodules reduced (in prominence), or nodules absent (Figure 4). We had a similar size range in the reference collection for both species, but not the largest sizes recorded for either species (Figure 5) (Robins & Ray, 1986). Nevertheless, bottlenose dolphins are unlikely to prey on fish at the upper size limit of these species (>400 mm, Gannon & Waples, 2004; Robins & Ray, 1986) so we would not expect to find those older fish in stomachs. Using only data from reference‐collection otoliths, we conducted a recursive partition analysis (i.e., Classification and Regression Tree [CART]) (SAS JMP 12) that produces binary splits of observations with similar response values into clusters (Breiman, Friedman, Stone, & Olshen, 1984). We used two candidate covariates, CL:SL and nodule category, to predict response variable spotted seatrout or weakfish. With this approach, all possible splits of candidate covariates create an optimal decision tree with the minimum number of misclassifications (maximizing homogeneity) while also minimizing the complexity, in this case using 10‐fold cross‐validation. The results from the CART analysis were used to assign spotted seatrout or weakfish prey otoliths to species. Comparisons were made between visual identification and assigned identification from the CART analysis. When species identification differed, two readers verified prey identification based on quantitative diagnostics of CL:SL or visual comparison with reference‐collection otoliths.

### 2.3 Estimating prey size

Using reference‐collection otoliths for croaker, spot, spotted seatrout, and weakfish, linear and curvilinear (Gompertz, Logistic) regression models were fitted to examine the relationship between the predictors (OL, SL, CL) and two measures of fish size, length (total length, mm), and weight (g) (SAS JMP 12). We assumed that the broad range in fish sizes available for each species would account for any differential growth among the predictors with fish growth (Aguirre, 2003). The best fit was determined by the lowest Akaike information criterion (AIC) values with two caveats. First, we used the simpler linear model when its ∆AIC ≤ 2 from the lowest AIC (Burnham & Anderson, 2004). Second, for consistency, if one model fit better for two of three predictors for a species, we used that model for all predictors. For the final models, we assessed how the variance among predictors affected estimated prey size for each species. First, we subtracted the measured fish size from the predicted fish size per fish for each of the predictors, OL, SL, and CL (referred to as measured–predicted). For each species, we used ANOVAs to test for differences in the mean measured–predicted across OL, SL, and CL for length, and separately for weight.

### 2.4 Assessing differential erosion rates

Detecting differential erosion rates across the predictors, otolith, sulcus, and cauda lengths, is confounded because it is not possible to know their initial lengths before a fish was eaten. From uneroded (i.e., reference collection) otoliths, however, we can model the expected relationship between pairs of predictors (baseline relationships), and determine if those relationships are maintained for eroded prey otoliths. We used two metrics to assess if digestive erosion affected lengths of the predictors equally. First, we calculated baseline relationships from reference‐collection otoliths for all pairs of predictors using linear regressions: OL to CL, OL to SL, and SL to CL. We then repeated the regressions for prey otoliths by grade and compared the direction and variability of the relationships to the baseline. For example, we overlaid the measured OLs from prey otoliths against the measured CLs from prey otoliths by grade category with the comparable baseline relationships. This series of plots was repeated for SL against CL and for OL against SL.

Given the strong relationship between each predictor and fish size, the second metric was to determine if differential rates of erosion on the three predictors significantly affected estimated fish size (length and weight). The equations from the best‐fitting models per predictor from reference‐collection otoliths were applied to estimate length and weight of fish from prey otoliths. We tested whether estimated prey size differed among measured predictors and between grade categories (Grade 0–2 and Grade 3–5) using a nested repeated measures analysis of variance (ANOVA) that incorporates a linear mixed‐effects model (JMP 12 with add‐in available from https://community.jmp.com/t5/JMP-Add-Ins/Full-Factorial-Repeated-Measures-ANOVA-Add-In/ta-p/23904). Repeated‐measures tests take into account that there were three measurements from each otolith, OL, SL, and CL. The resulting three estimated prey sizes were nested within their corresponding grade category to account for variation between grade categories. These analyses were performed separately for length and weight, and separately for croaker, spot, and weakfish. Data from spotted seatrout were not included because few otoliths were recovered from stomachs (Table 1). Tukey HSD post hoc tests were used when ANOVAs were significant.

**Table 1:** Number of fish as represented by prey otoliths recovered from stomachs of 120 stranded common bottlenose dolphins (*Tursiops truncatus*). Otoliths were graded by digestive erosion: 0, undamaged; 1, barely degraded, sulcus fully visible; 2, slightly degraded, somewhat chalky appearance, sulcus is still fully visible; 3–5, moderately to severely degraded, erosion affecting sulcus edge, or broken. See Figure 2. The four species we examined were croaker (*Micropogonias undulatus*), spot (*Leiostomus xanthurus*), spotted seatrout (*Cynoscion nebulosus*), and weakfish (*Cynoscion regalis*). All other species were combined

Common Name	Grade 0	Grade 1	Grade 2	Grade 3–5	All grades
Croaker	4	19	261	1,727	2,011
Spot	0	4	191	1,976	2,171
Spotted seatrout	0	0	3	22	25
Weakfish	0	20	97	984	1,101
Other species	0	17	191	1,604	1,812
Total	4	60	743	6,313	7,120

### 2.5 Methods of estimating consumption biomass

One of the assumptions of applying the mean estimated fish length or weight from otoliths Grade 0–2 across all samples to otoliths Grade 3–5 is that there are no seasonal differences in prey size. We tested this assumption using weight because it is used to examine consumption biomass by a predator. First, we estimated weights of fish represented by prey otoliths using species‐specific predictors (OL, SL, or CL) depending on results from the nested repeated measures ANOVA outlined above. We then tested for differences in mean weight across seasons within a given species and grade category using a nonparametric Kruskal–Wallis test and Tukey–Kramer HSD post hoc test because parametric test assumptions were violated (unequal variances, *p* < .05). For example, differences in mean weight among seasons for croaker prey otoliths Grade 0–2 were tested separately from differences in mean weight among seasons for croaker prey otoliths Grade 3–5. Similarly, these tests were repeated for spot and weakfish.

Lastly, we examined differences in consumption biomass of a given prey species for each dolphin across three scenarios that exclude or include measurements of otoliths Grade 3–5:

#### 2.5.1 Standard scenario

Only fish weights estimated from otoliths Grade 0–2 were used. Mean estimated fish weight was calculated from otoliths Grade 0–2 across all dolphins and then applied to the number of otoliths Grade 3–5 for each dolphin.

$${\text{Biomass}}_{\text{St}}=\sum W_{\text{Grade}0-2_{\text{St}}}+N_{\text{Grade}3-5_{\text{St}}}\left(\frac{\sum W_{\text{Grade}0-2}}{N_{\text{Grade}0-2}}\right);$$

where Biomass is the weight of all fish of a given species found within an individual dolphin's stomach (_St_), *W* is the estimated weight from recovered otoliths, *N* is the number of otoliths, and subscripts refer to grade categories (i.e., _Grade0–2_ and _Grade3–5_).

#### 2.5.2 Iterative scenario

Only fish weights estimated from otoliths Grade 0–2 were used. In contrast to Scenario A, the mean estimated fish weight calculated from otoliths Grade 0–2 was applied to otoliths Grade 3–5 in an iterative process. When possible, the mean estimated weight from otoliths Grade 0–2 within a stomach was applied to otoliths Grade 3–5 within that same stomach (Gannon & Waples, 2004).

$${\text{Biomass}}_{\text{St}}=\sum W_{\text{Grade}0-2_{\text{St}}}+N_{\text{Grade}3-5_{\text{St}}}\left(\frac{\sum W_{\text{Grade}0-2_{\text{St}}}}{N_{\text{Grade}0-2_{\text{St}}}}\right).$$

If there were no otoliths Grade 0–2 within the stomach, the mean estimated weight of otoliths Grade 0–2 from stomachs of dolphins that stranded within the same month was applied to otoliths Grade 3–5.

$${\text{Biomass}}_{\text{St}}=\sum W_{\text{Grade}0-2_{\text{St}}}+N_{\text{Grade}3-5_{\text{St}}}\left(\frac{\sum W_{\text{Grade}0-2_{M}}}{N_{\text{Grade}0-2_{M}}}\right);$$

where subsubscript _M_ refers to month.

If there were no otoliths Grade 0–2 within the month, the mean estimated weight of otoliths Grade 0–2 from stomachs of dolphins that stranded within the same season was applied to otoliths Grade 3–5.

$${\text{Biomass}}_{\text{St}}=\sum W_{\text{Grade}0-2_{\text{St}}}+N_{\text{Grade}3-5_{\text{St}}}\left(\frac{\sum W_{\text{Grade}0-2_{\text{Se}}}}{N_{\text{Grade}0-2_{\text{Se}}}}\right);$$

where subsubscript _Se _refers to season.

If there were no otoliths Grade 0–2 within the same season, the mean estimated weight of otoliths Grade 0–2 from stomachs across the study was applied to otoliths Grade 3–5 (essentially the Standard Scenario).

#### 2.5.3 Inclusive scenario

In this analysis, estimated fish weights from all otoliths, Grade 0–2 and Grade 3–5, were used. The mean estimated fish weight was calculated from otoliths (all grades) in an iterative process similar to the Iterative Scenario to apply to unmeasured otoliths (i.e., broken or not subsampled for measurement).

$${\text{Biomass}}_{\text{St}}=\sum W_{{\text{Measured}}_{\text{St}}}+N_{{\text{Unmeasured}}_{\text{St}}}\left(\frac{\sum W_{{\text{Measured}}_{\text{St, M, Se}}}}{N_{{\text{Measured}}_{\text{St, M, Se}}}}\right);$$

where subscripts refer to all measured otoliths (_Measured_), or unmeasured otoliths (_Unmeasured_).

Using the estimated consumed biomass of each prey species across the three scenarios for each dolphin, we tested for differences using a repeated measures ANOVA and Tukey HSD post hoc test (JMP 12). These analyses were performed for all species except spotted seatrout because of a low sample size.

## 3 RESULTS

### 3.1 Sampling

The reference collection included croaker, spot, spotted seatrout, and weakfish specimens that were all collected in NC. Specimens were distributed across the size range reportedly consumed by bottlenose dolphins in NC (Figure 5) (Gannon & Waples, 2004). Weight data were missing for some spotted seatrout and weakfish specimens resulting in lower samples sizes used in the regression analyses than the CART analysis. Croaker had the lowest sample size (*n* = 53) and most constrained size range.

Out of 213 stranded bottlenose dolphins, 169 met criteria for inclusion in the analysis. Of those, 120 had identifiable contents in the forestomach, esophagus, or both. All age‐class categories were represented: YOY (*n* = 6), older calves (*n* = 33), subadults (*n* = 34), and adult (*n* = 36). Age‐class category was unknown for 11 dolphins because length could not be determined, primarily because their flukes were removed during human interactions (see Byrd et al., 2014). The 120 dolphins were distributed across all seasons: Spring, *n* = 44; Summer, *n* = 16; Fall, *n* = 25; Winter, *n* = 35. Croaker otoliths were found in 52% of the 120 stomachs, spot in 62%, spotted seatrout in 10%, and weakfish in 47%. These four species comprised 74% (*n* = 5,308 of 7,120) of fish represented by otoliths (Table 1). The majority of prey otoliths were Grade 3–5 (89%) (Table 1). Few prey otoliths removed from fish skulls (*n* = 386 of 5,308) were undamaged (Grade 0, *n* = 4) or barely degraded (Grade 1, *n* = 39) because most skulls had been partially digested. The majority of otoliths recovered from such skulls were Grade 2 (*n* = 159) or Grade 3–5 (*n* = 184).

### 3.2 Discrimination between spotted seatrout and weakfish

The CART analysis using reference‐collection otoliths indicated that one split produced the optimal decision tree using the predictor variable CL:SL at a cut‐point of .49. With one split, the *r* ^2^ = .89 and the classification accuracy was .98 for both species. All otoliths with CL:SL < .49 were assigned to spotted seatrout (*n* = 164). All but six otoliths with CL:SL > .49 were assigned to weakfish (*n* = 173). Forcing an additional split showed that the uncertainty occurred when CL:SL was between .49 and .50, which included six spotted seatrout and 25 weakfish (*r* ^2^ = .93) (Table 2). The contribution of nodules was not a candidate in either the optimal split or the forced extra split.

**Table 2:** Predicted species, spotted seatrout (*Cynoscion nebulosus)* or weakfish (*Cynoscion regalis*), assignment from a recursive partition analysis of Cauda Length (CL): Sulcus Length (SL) from reference‐collection otoliths. With this analysis, probabilities are never zero (*p* ≠ .0) even when the assignment number (*n*) is 0

Species	CL:SL		CL:SL		CL:SL	
	≤ 0.48		0.49 < 0.50		≥ 0.51	
	*n*	*p*	*n*	*p*	*n*	*p*
Spotted seatrout	164	.997	6	.202	0	.003
Weakfish	0	.003	25	.798	142	.997

The cut‐points of <.49 and >.50 were applied to unbroken prey otoliths initially identified as 125 spotted seatrout and 914 weakfish, resulting in a predicted distribution of 17 spotted seatrout (<.49), 1,001 weakfish (>.50), and 21 fish that fell between the two cut‐points. All 17 spotted seatrout were initially correctly identified. Of the 1,001 fish predicted to be weakfish, 99 were originally identified as spotted seatrout. Of the 21 fish with CL:SL values from .49 to .50, initial identifications were 9 spotted seatrout and 12 weakfish. The 21 were re‐examined by visual comparison to reference‐collection otoliths of similar size resulting in changes to the identification (6 spotted seatrout, 15 weakfish), which is slightly different than applying the CART analysis probability of .80 (4 spotted seatrout, 17 weakfish). Although CL:SL could not be measured and calculated for broken otoliths initially identified as seven spotted seatrout and 80 weakfish, they were visually compared to reference‐collection otoliths for the second time. Five of the seven otoliths originally as spotted seatrout were changed to weakfish resulting in a final count of two spotted seatrout and 85 weakfish. Final identifications for measured and broken otoliths were 25 spotted seatrout (23 measured and 2 broken) and 1,101 weakfish (1,015 measured and 85 broken). Nine of the 12 dolphins that ate spotted seatrout also ate weakfish.

Nodules on reference‐collection otoliths were present for 96% of spotted seatrout (108 of 113) and 34% of weakfish (37 of 110) (Figure 4). When present, nodules were categorized as reduced for 31% of spotted seatrout and 57% of weakfish. Despite digestive erosion, nodules were present and not reduced on most prey otoliths of spotted seatrout (present = 23, absent = 1, not recorded/broken = 2). Weakfish showed the opposite trend (present = 190, reduced = 97, absent = 734, not recorded/broken = 80).

### 3.3 Estimating prey size

**Figure A1:**
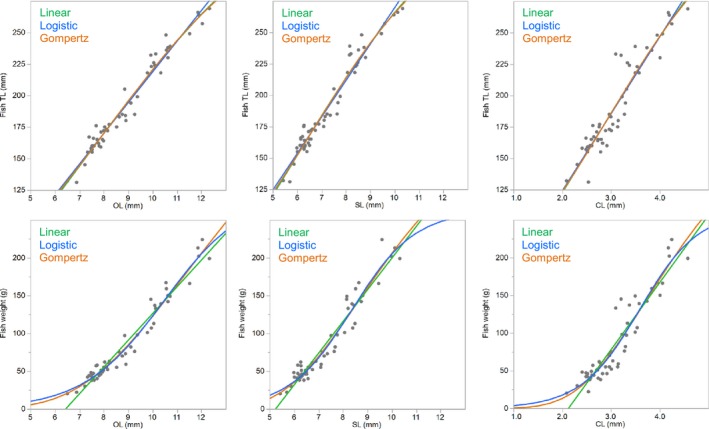
For croaker, Linear (green line), Logistic (blue line), and Gompertz (orange line) models were compared using ∆Akaike Information Criterion (∆AIC) and r^2^ values to determine the best fit between otolith length (OL), sulcus length (SL), and cauda length (CL), to fish total length (Fish TL, top row) and fish weight (bottom row). See Supplemental Tables A1 & A2 for ∆AIC and r^2^ values.

**Figure A2:**
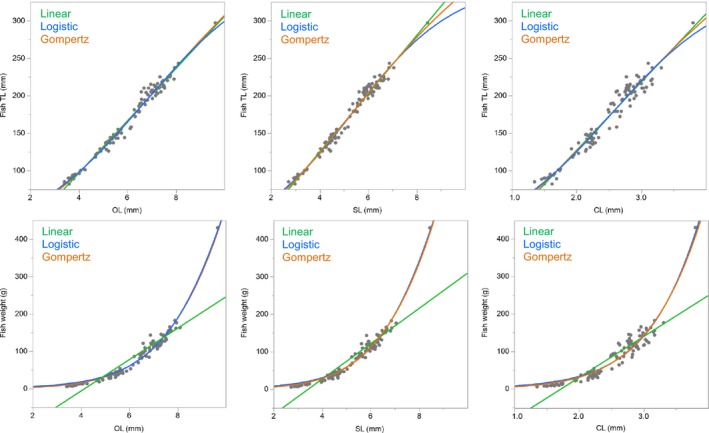
For spot, Linear (green line), Logistic (blue line), and Gompertz (orange line) models were compared using ∆Akaike Information Criterion (∆AIC) and r^2^ values to determine the best fit between otolith length (OL), sulcus length (SL), and cauda length (CL), to fish total length (Fish TL, top row) and fish weight (bottom row). See Supplemental Tables A1 & A2 for ∆AIC and r^2^ values.

**Figure A3:**
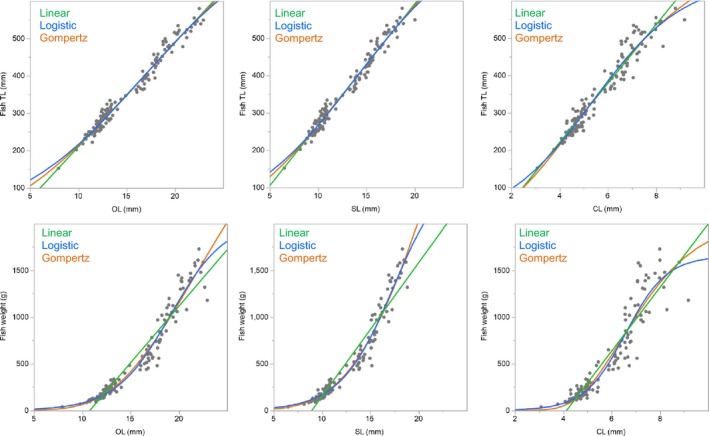
For spotted seatrout, Linear (green line), Logistic (blue line), and Gompertz (orange line) models were compared using ∆Akaike Information Criterion (∆AIC) and r^2^ values to determine the best fit between otolith length (OL), sulcus length (SL), and cauda length (CL), to fish total length (Fish TL, top row) and fish weight (bottom row). See Supplemental Tables A1 & A2 for ∆AIC and r^2^ values.

**Figure A4:**
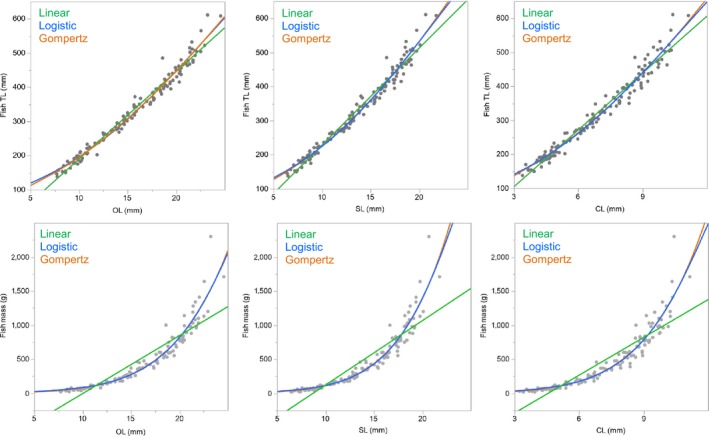
For weakfish, Linear (green line), Logistic (blue line), and Gompertz (orange line) models were compared using ∆Akaike Information Criterion (∆AIC) and r^2^ values to determine the best fit between otolith length (OL), sulcus length (SL), and cauda length (CL), to fish total length (Fish TL, top row) and fish weight (bottom row). See Supplemental Tables A1 & A2 for ∆AIC and r^2^ values.

Using data from reference‐collection otoliths, the best‐fitting model to estimate prey size differed among species and between predicting length versus weight (Table A1). The ∆AIC values indicated that nonlinear models often provided the best fit across the three predictive features (Figures A1, A2, A3, A4, Table A1). For length models of spotted seatrout, the best‐fitting models differed among the three predictors; models from OL and SL were linear (∆AIC = 0), but the ∆AIC value for the linear model from CL was 12.5. As a compromise, we selected the logistic model for which the CL model ∆AIC = 0, and the OL and SL model ∆AIC values were slightly over 2.0 (2.1 and 2.3, respectively). Of the best models for each species and predictor, *r* ^2^ values were high, ranging from .88 to .98 (Table 3). Differences in *r* ^2^ values among predictors were negligible in most cases; however, for croaker the *r* ^2^ values of models predicting length and weight from CL (length = .88, weight = .89) were .08 lower than from OL (length = .96, weight = .97) (Table 3). Visual inspection of the residuals showed no trends meaning lower *r* ^2^ values represented higher variances around estimated fish size, but no bias in the direction of that variance. For each species, there was no significant difference in the mean measured–predicted values for length and for weight based on OL, SL, and CL (croaker TL: *df* = 2, *F* ratio < .0001, *p* = 1.0; croaker FM: *df* = 2, *F* ratio = .0013, *p* = 1.0; spot TL: *df* = 2, *F* ratio = .0002, *p* = 1.0; spot FM: *df* = 2, *F* ratio = .0085, *p* = .99; spotted seatrout TL: *df* = 2, *F* ratio = .0004, *p* = 1.0; spotted seatrout FM: *df* = 2, *F* ratio = .0083, *p* = .99; weakfish TL: *df* = 2, *F* ratio < .0001, *p* = 1.0, weakfish FM: *df* = 2, *F* ratio = .0015, *p* = 1.0).

**Table 3:** Best‐fitting models used to predict the relationship between otolith length (OL), sulcus length (SL), and cauda length (CL) (mm) to fish total length (FTL, mm) and fish weight (FW, g) for croaker (*Micropogonias undulatus)*, spot (*Leiostomus xanthurus*), spotted seatrout (*Cynoscion nebulosus*), weakfish (*Cynoscion regalis*). Gompertz and Logistic models have three parameters. Regression = Regress. EXP = inverse natural logarithm

Species	Regress.	Model	*r* ^2^	Prediction Model
Croaker	OL‐FTL	Linear	.96	FTL = (−25.51784) + (24.410412*OL)
	SL‐FTL	Linear	.95	FTL = (−20.75988) + (29.028549*SL)
	CL‐FTL	Linear	.88	FTL = (1.2494378) + (61.30351*CL)
	OL‐FW	Logistic	.97	FW = 286.38443/(1 + EXP(−0.6072569*(OL−10.473646)))
	SL‐FW	Logistic	.94	FW = 262.50792/(1 + EXP(−0.7774777*(SL−8.3679234)))
	CL‐FW	Logistic	.89	FW = 261.82446/(1 + EXP(−1.6664716*(CL−3.5895675)))
Spot	OL‐FTL	Logistic	.98	FTL = 380.79763/(1 + EXP(−0.3929095*(OL−6.705528)))
	SL‐FTL	Logistic	.98	FTL = 353.24343/(1 + EXP(−0.4677786*(SL−5.3485423)))
	CL‐FTL	Logistic	.95	FTL = 350.7459/(1 + EXP(−1.1056006*(CL−2.5360011)))
	OL‐FW	Gompertz	.97	FW = 85,841.563*(EXP(−EXP(−0.085671*(OL−29.16622)))
	SL‐FW	Gompertz	.96	FW = 29,777.77*(EXP(‐EXP(−0.1101145*(SL−21.640302))))
	CL‐FW	Gompertz	.92	FW = 483,412.11*(EXP(‐EXP(−0.1733285*(CL−15.095524))))
Spotted seatrout	OL‐FTL	Logistic	.97	FTL = 792.34349/(1 + EXP(−0.1466631*(OL−16.694034)))
	SL‐FTL	Logistic	.97	FTL = 793.22148/(1 + EXP(−0.1702467*(SL−14.004909)))
	CL‐FTL	Logistic	.94	FTL = 659.58373/(1 + EXP(−0.5267355*(CL−5.3660654)))
	OL‐FW	Logistic	.95	FW = 2042.2502/(1 + EXP(−0.3516577*(OL−19.132198)))
	SL‐FW	Logistic	.95	FW = 2029.3521/(1 + EXP(−0.4105468*(SL−16.059515)))
	CL‐FW	Logistic	.90	FW = 1654.0488/(1 + EXP(−1.1770936*(CL−6.5814683)))
Weakfish	OL‐FTL	Gompertz	.98	FTL = 2,477.0097*(EXP(−EXP(−0.0394058*(OL−33.676056))))
	SL‐FTL	Gompertz	.97	FTL = 3,409.5556*(EXP(‐EXP(−0.0379836*(SL−36.322885))))
	CL‐FTL	Gompertz	.97	FTL = 2,957.7632*(EXP(‐EXP(−0.0793614*(CL−17.141034))))
	OL‐FW	Logistic	.93	FW = 5,861.9572/(1 + EXP(−0.2409874*(OL−27.556653)))
	SL‐FW	Logistic	.93	FW = 5,941.1188/(1 + EXP(−0.2737339*(SL−24.319011)))
	CL‐FW	Logistic	.92	FW = 5,377.982/(1 + EXP(−0.5400035*(CL−12.275319)))

### 3.4 Assessing differential erosion rates

**Figure 6:**
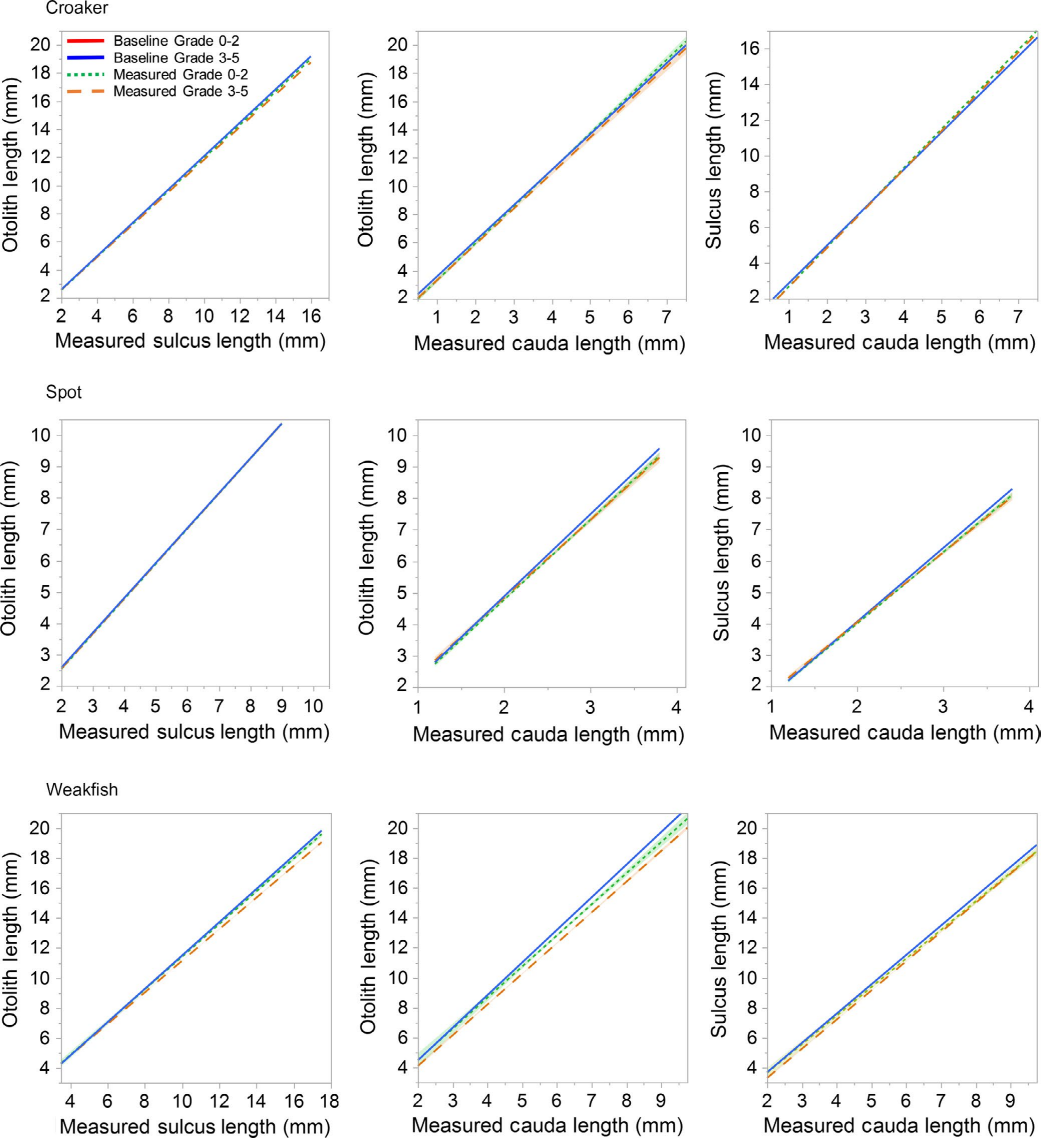
Pair‐wise relationships between otolith length (OL), sulcus length (SL), and caudal length (CL) measured for prey otoliths from croaker (*Micropogonias undulatus*), spot (*Leiostomus xanthurus*), and weakfish (*Cynoscion regalis*) relative to predicted baseline relationships modeled from reference‐collection otoliths. The x‐axes represent measured lengths from prey otoliths. Each plot includes four regressions: baseline predicted length for otoliths Grade 0–2, baseline predicted length for otoliths Grade 3–5, measured length for otoliths Grade 0–2, measured length for otoliths Grade 3–5; however, the baseline plots for each grade category are always overlaid. The 95% confidence intervals around the means are shown as shaded intervals, but some are too narrow to be visible

Relationships between predictors (OL, SL, and CL) measured from prey otoliths showed deviation from the baseline relationships, although analyses of covariance (ANCOVA) tests could not be used because slopes of the regression lines were unequal (*p* < .05). For spot and weakfish, regressions of measured OL to measured CL and measured SL to measured CL were negatively biased relative to the expected 1:1 baseline relationship (Figure 6). Grade category did not affect the degree of negative bias for spot or SL to CL for weakfish, but did for OL to CL for weakfish. Otolith length to SL, however, showed no bias between measured and baseline relationships.

Regression results from croaker otoliths differed from spot and weakfish. For croaker, regressions of measured OL to measured CL for grade category 0–2 became positively biased with increased measured CL relative to the baseline relationship (Figure 6). A similar pattern was seen for regression lines of SL to CL for both grade categories. Finally, regressions of measured OL to measured SL were negatively biased relative to the baseline relationship, more so for Grade 3–5 otoliths than Grade 0–2 otoliths.

**Figure 7:**
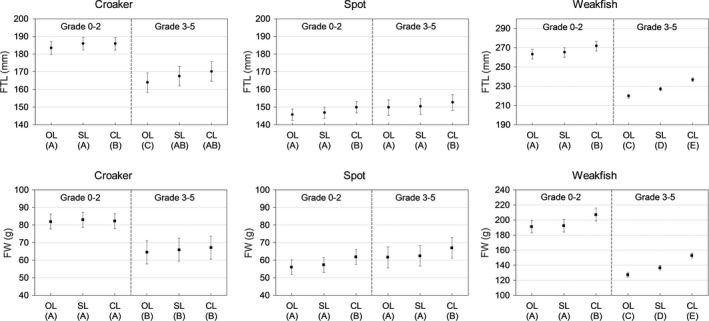
For prey otoliths removed from stranded bottlenose dolphins (*Tursiops truncatus*), mean estimated fish total length and weight from predictors otolith length (OL), sulcus length (SL), and cauda length (CL) nested within grade categories, Grade 0–2 (no to slight digestive erosion) and Grade 3–5 (moderate to severe digestive erosion). Beneath x‐labels are letters denoting significant differences identified from Tukey HSD post hoc comparisons for significant nested repeated measures ANOVAs. Levels with different letters are significantly different. Standard error bars are shown. Note that axes for weakfish (*Cynoscion regalis*) are different from croaker (*Micropogonias undulatus*) and spot (*Leiostomus xanthurus*)

The differential erosion rates seen in the afore‐mentioned baseline model comparisons were evident in the estimated fish sizes across the three predictors, OL, SL, and CL. For estimating original fish size from prey otoliths, the most common pattern was that estimated fish size (length and weight) was generally larger from CL than SL and/or OL (Table 4, Figure 7). For spot, there was no significant difference in estimated length and weight between grade categories, but CL did estimate larger prey size than OL and SL. Across both grade categories, estimated size from CL was ~ 2.5% larger than from OL for length and ~9% larger for weight. For weakfish, there was a significant difference in estimated length and weight among predictors and across grade categories. Weakfish CL always estimated significantly larger fish than SL and OL (*p* < .05) and otoliths Grade 0–2 estimated significantly larger fish than otoliths Grade 3–5 (*p* < .05). In fact, estimated length from otoliths Grade 0–2 was 3% larger using CL rather than OL, and estimated weight from otoliths Grade 0–2 were 8% larger using CL rather than OL. The difference between the two predictors was even larger for otoliths Grade 3–5 (~8% for length and 20% for weight).

**Table 4:** Results of nested repeated measures ANOVAs by species comparing estimated fish total length and weight among predictors (pred.), otolith length [OL], sulcus length [SL], cauda length [CL], nested within otolith grade categories (Grade cat.): 0–2 and 3–5. Signficant *p* values are italicized. Species are croaker (*Micropogonias undulatus*), spot (*Leiostomus xanthurus*), spotted seatrout (*Cynoscion nebulosus*), and weakfish (*Cynoscion regalis*)

Total length				Weight			
Source	*df*	*F* Ratio	*p* > *F*	Source	*df*	*F* Ratio	*p* > *F*
Croaker (Grade 0–2 = 284, Grade 3–5 = 120)							
Grade cat.	1	7.48	*.0065*	Grade cat.	1	4.57	*.0332*
Pred.	2	30.02	*<.0001*	Pred.	2	2.47	.0855
Grade cat. * Pred.	2	5.53	*.0041*	Grade cat. * Pred.	2	1.6	.2025
Spot (Grade 0–2 = 195, Grade 3–5 = 101)							
Grade cat.	1	0.39	.5341	Grade cat.	1	0.55	.4607
Pred.	2	36.01	*<.0001*	Pred.	2	30.56	*<.0001*
Grade cat. * Pred.	2	1.31	.2713	Grade cat. * Pred.	2	0.05	.9469
Weakfish (Grade 0–2 = 117, Grade 3–5 = 876)							
Grade cat.	1	55.44	*<.0001*	Grade cat.	1	42.28	*<.0001*
Pred.	2	441.69	*<.0001*	Pred.	2	205.30	*<.0001*
Grade cat. * Pred.	2	51.59	*<.0001*	Grade cat. * Pred.	2	11.32	*<.0001*

The results for croaker were less consistent between estimated length and weight. For estimated length of croaker, there was a significant difference (*p* < .05) among predictors and across grade categories (Table 4). Croaker CL and SL estimated larger lengths than OL, but there was no difference in estimated length between SL and CL. Mean estimated length was generally larger from otoliths Grade 0–2 than Grade 3–5, but this was most pronounced for the significantly lower estimated length from OL. For estimated weight of croaker, there was no significant difference among predictors, but otoliths Grade 0–2 estimated significantly larger weights (25%) than otoliths Grade 3–5 (*p* < .05).

The differential digestive erosion rates across the three predictors could be caused by erosion decreasing OL relative to CL or SL, or erosion increasing CL or SL relative to the other predictors. Our inspection of reference‐collection otoliths found that for all three species the sulcus walls were nearly parallel except for a slight angle inward at the sulcus floor where the transition to the walls was smooth. The shape of the sulcus walls means that erosion would not increase CL and SL; thus, erosion was greater for OL where the edges are abraded than for CL or SL, as the latter two were contained within the margin of the otolith. For spot and weakfish, for which the ostium portion of the sulcus is close to the otolith edge, the effect of digestion was greater for SL than for CL.

### 3.5 Methods of estimating consumption biomass

**Figure 8:**
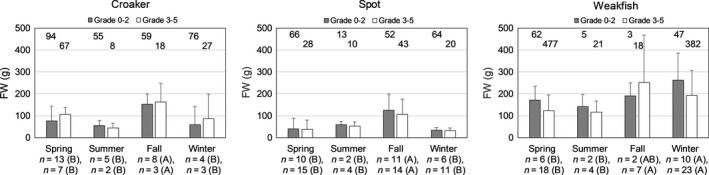
For prey otoliths removed from stranded bottlenose dolphin (*Tursiops truncatus*), mean estimated fish weight (g) by season and grade category of croaker (*Micropogonias undulatus*) estimated from sulcus length, and spot (*Leiostomus xanthurus*) and weakfish (*Cynoscion regalis*) estimated from cauda length. Error bars show one standard deviation; otolith sample size is shown at the top of each graph. On the *x*‐axes under season are numbers of dolphins from which the otoliths were recovered and letters (A and B) denoting significant differences among seasons within a grade category with data for Grade 0–2 listed above Grade 3–5. Numbers of dolphins are not additive across prey species

There were significant differences in mean estimated weight across seasons for otoliths Grade 0–2 and Grade 3–5 from croaker, spot, and weakfish (Figure 8). Mean estimated weights of croaker and spot were significantly larger during fall than during other seasons for otoliths Grade 0–2 (croaker: *df* = 3, *F* Ratio = 32.85, *p* < .0001; spot: *df* = 3, *F* Ratio = 42.36, *p* < .0001) and otoliths Grade 3–5 (croaker: *df* = 3, *F* Ratio = 19.05, *p* < .0001; spot: *df* = 3, *F* Ratio = 14.62, *p* < .0001). For weakfish otoliths Grade 0–2, estimated fish weight was significantly greater during winter than during spring and summer (*df* = 3, *F* Ratio = 9.56, *p* < .0001). For weakfish otoliths Grade 3–5, estimated fish weights during winter and fall were significantly greater than during spring and summer (*df* = 3, *F* Ratio = 42.72, *p* < .0001).

**Figure 9:**
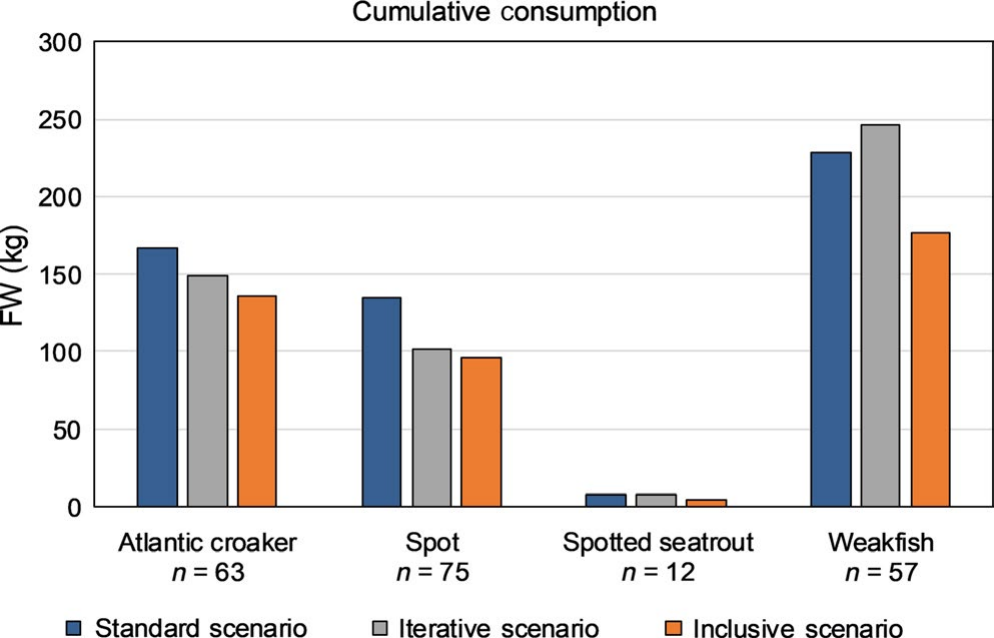
Cumulative estimated fish weight consumed by stranded dolphins across three scenarios (Standard, Iterative, Inclusive) that estimate weight from otoliths Grade 3–5 differently. Sample size under each prey species (croaker [*Micropogonias undulatus*], spot [*Leiostomus xanthurus*], spotted seatrout [*Cynoscion nebulosus*], and weakfish [*Cynoscion regalis*]) is the number of stranded bottlenose dolphins containing that species (out of 120 dolphins)

Estimated weights across the three scenarios for estimating consumption biomass were significantly different for weakfish (*df* = 2, *F* Ratio = 6.90, *p* = .0015), but no significant difference was found for croaker (*df* = 2, *F* Ratio = 0.81, *p* = .45) or spot (*df* = 2, *F* Ratio = 2.66, *p* = .07) (Figure 9). For weakfish, weights calculated in the Inclusive Scenario (using actual measurements from otoliths Grade 3–5) were significantly lower than the Standard and Iterative Scenarios. For the iterative selection approach (Iterative and Inclusive Scenarios), we found otoliths Grade 0–2 within the stomach, month, or season. Most stomachs containing weakfish (38 of 57) had otoliths Grade 0–2 within the stomach to scale to the otoliths Grade 3–5, while less than half of the stomachs containing croaker (30 of 63) and spot (32 of 75) had otoliths Grade 0–2 within the same stomach with otoliths Grade 3–5. Many stomachs had otoliths Grade 3–5 that could be scaled with otoliths Grade 0–2 from other stomachs in the same month; relatively few stomachs had otoliths Grade 3–5 that could only be scaled with otoliths Grade 0–2 from the same season (croaker = 5, spot = 2, weakfish = 13).

## 4 DISCUSSION

Use of the sulcus provided benefits of producing an easily repeatable way to distinguish between species with similar otoliths and of producing estimates of fish size with a reduced influence of digestion. Qualitative and quantitative assessments of the sulcus have been used in nondiet studies to distinguish among genera, species, and stocks (Chao, 1978; Torres et al., 2000; Tuset et al., 2008). The quantitative approach used in the current study was simple compared to more involved otolith shape analyses used in fish studies (Campana & Casselman, 1993; Colura & King, 1995; Škeljo & Ferri, 2012). Otolith chemistry is an alternative approach that has been reported for distinguishing among fish stocks in fish‐focused studies (e.g., Campana, Chouinard, Hanson, Fréchet, & Brattey, 2000) and among species in a pinniped diet study (Ferenbaugh, Strauss, Tollit, Chen, & Diamond, 2009; Kemp, Swearer, Jenkins, & Robertson, 2011); however, these analyses come at a greater monetary and time expense. Our simpler approach was still able to confirm spotted seatrout predation by bottlenose dolphins in NC, a result not found by Gannon and Waples (2004) in a study from the same geographic area. Given the limited number of spotted seatrout otoliths recovered in the current study, it is possible that the species was not represented by otoliths in Gannon and Waples (2004) or that they were not identified when using qualitative visual methods. The current study also found otoliths from both species in the same stomach, illustrating how easy it could be to assign them all to one species or another. Using this approach in other diet studies for predators of spotted seatrout and weakfish would reduce uncertainty in identification where the two species overlap (Atlantic) and where weakfish has been reported outside of its known range (Gulf of Mexico) (Bowen, 2011). Additionally, the approach could be adapted to incorporate other congeners of weakfish and spotted seatrout or adapted to other groups of species.

Understanding diets of piscivorous predators and the connection to predation effects on prey is influenced by the digestive erosion of otoliths that affects the size and, for fragile otoliths especially, number recovered (Bowen & Iverson, 2013; Casaux, Favero, Barrera‐Oro, & Silva, 1995; Johnson, Ross, McKenna, & Lewis, 2006; Pierce & Boyle, 1991; Tollit et al., 1997). In the current study, comparisons of measured predictors from prey otoliths to baseline relationships between predictors indicated differential erosion rates for OL, SL, and CL. These differences were borne out in the significant differences for spot and weakfish in estimated fish length and weight among the predictors, and for croaker in estimated fish length among predictors. For all species, where significant differences occurred, estimated fish size from OL was smaller than CL, but often the same as SL. The models to estimate fish size from predictors had similar and high explanatory powers and did not account for differences in estimated prey size from these predictors in prey otoliths. As such, differences in estimated size across predictors indicate that erosion differentially affected OL compared to CL and sometimes SL. Although digestive erosion would affect otoliths in all dimensions, the margins of the sulcus (i.e., sulcus and cauda lengths) in the three species we examined are not expected to change much with erosion given that the sulcus walls are nearly parallel and not angled away from the otolith edges. The significantly larger estimates of fish size from CL or SL, therefore, are likely to be closer to the original size of the fish consumed.

**Figure A5:**
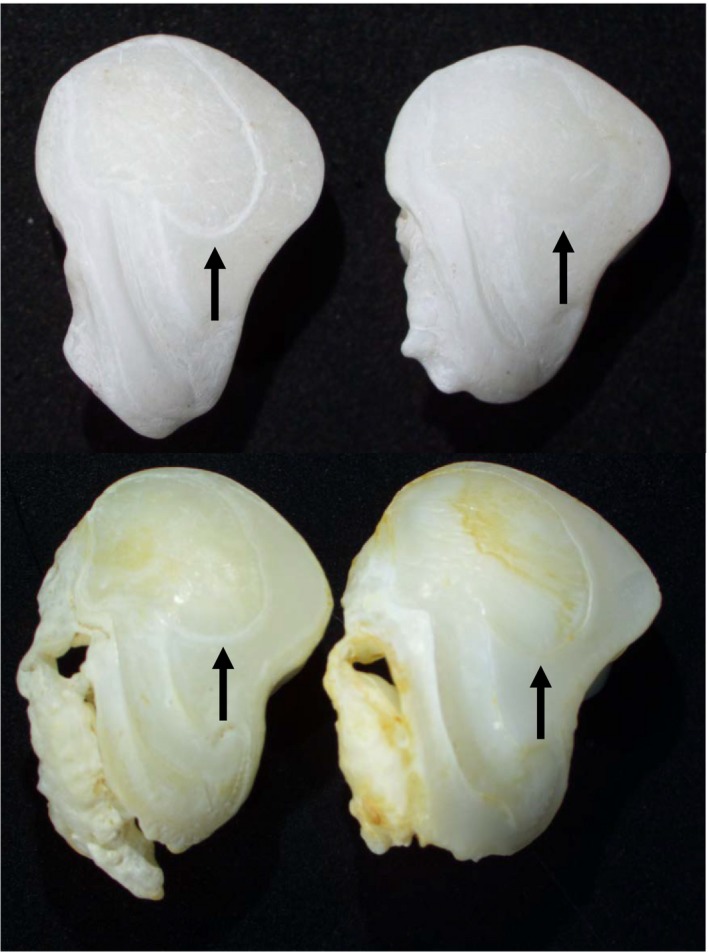
Croaker otoliths removed from bottlenose dolphin stomachs (top: grade 2, bottom: grade 1). Notice variation in the shape and inferior position of the ostium (arrows).

In the absence of correction factors, the current study demonstrated that using alternatives to OL, such as CL or SL, to estimate fish size is a reasonable and time‐efficient method to limit erosion bias. For spot, weakfish, and spotted seatrout, the ostium edge is close to the otolith edge making CL a better predictor of fish size than SL. In fact, the CL for weakfish was visibly affected in only the most eroded (Grade 5) of otoliths (Figure 2). For croaker, there was notable variability in sulcus shape especially at the inferior point of the ostium (Figure A5), increasing variability for the relationship between CL and the other predictive features, and decreasing the difference in estimated fish size between the SL and CL. Results for croaker may also be influenced by the lower sample size compared to spot and weakfish. The percent size difference between using OL and an alternative predictor for a given species and grade category in this study was less than from captive pinniped experiments (Reviewed in Bowen & Iverson, 2013). Although digestive erosion may not be directly comparable between otoliths recovered from pinnipeds (feces) and cetaceans (stomachs), this comparison indicates that our findings are within a reasonable range. The percent differences were small between estimated size from CL or SL compared to OL; however, the differences could translate into large effects on a fish stock when estimated consumed biomass is applied to, in this case, the total number of dolphins that seasonally occur in NC (>10,000; Hayes et al., 2018) (Laake, Browne, DeLong, & Huber, 2002).

It is still unknown how well the alternatives to OL compensate for erosion of otoliths with moderate to severe erosion (Grade 3–5). Although the mean estimated sizes for croaker and weakfish from otoliths Grade 3–5 were smaller than otoliths Grade 0–2, the opposite was true for spot, regardless of the predictor. Thus, it cannot be assumed that the mean size of otoliths Grade 0–2 is representative of otoliths Grade 3–5. For example, bottlenose dolphins along the US mid‐Atlantic are known to feed multiple times in a single day (Shippee, 2014). In this case, the stomach could have otoliths of different sizes from schools of differently sized fish. Otoliths from smaller fish of even the same species are likely to erode faster (Phillips & Harvey, 2009; Tollit et al., 1997), which could result in smaller fish being under‐represented by otoliths with little to no erosion. Without grade‐specific correction factors (Tollit et al., 1997), should intact otoliths with moderate to severe erosion be excluded altogether? On a dolphin by dolphin basis, a difference in estimated consumed biomass among scenarios was only significant for weakfish with the Inclusive Scenario resulting in smaller biomass estimates than the other scenarios. Although estimated size may be more accurate for otoliths with less erosion, studies often recover few in that condition. The resulting sample size limitation may mean that the least eroded otoliths are not representative of fish sizes across an entire study, especially given seasonal changes in size classes of fish recovered in this study and elsewhere (Johnson et al., 2006). The Iterative Scenario to estimate weight of prey species would account for the temporal changes in fish size and number available for consumption, and for variability among individual dolphins. We suggest that this is a preferable alternative to the Standard Scenario. Using alternative measurements to OL, such as CL for weakfish, may allow inclusion of some or all otoliths of moderate to severe erosion (Inclusive Scenario) by minimizing erosion bias. No approach is without bias, however. The chosen approach (i.e., scenario), therefore, may depend on a researcher's goal. For example, the Inclusive Scenario would provide the minimum estimated biomass. The Iterative Scenario, however, may be a better choice than the Inclusive Scenario if there is a need for a risk‐adverse approach to estimate total consumed biomass based on the prey's management status.

There are limitations and biases that should be acknowledged in the current study. Sciaenids, with their robust otoliths and distinct sulcuses, were ideal for testing the value of this approach for estimating diets, but this method has yet to be applied to species with small, fragile otoliths. Some species are poor candidates for using the lengths of the sulcus or the cauda because the entire sulcus extends to the otolith margin (Tuset et al., 2008), but sulcus widths may be an option to explore in such cases. Nevertheless, this approach may not work across all predators and prey, but may be broadly useful for bottlenose dolphins because of their tendency to prey on soniferous fishes (Barros, 1993; Barros & Odell, 1990; Barros & Wells, 1998; Gannon et al., 2005; Gannon & Waples, 2004; Pate & McFee, 2012), which often have robust otoliths (Cruz & Lombarte, 2004). Another possible bias is measurement error; however, we took steps in our methods to minimize this error. Finally, one consideration may be that using all or part of the sulcus to estimate weight of only some species may overestimate their importance in the diet relative to other species, especially if otoliths from other species are likely to be completely digested. In the current study, the four species considered accounted for 75% of all otoliths recovered, which probably limited bias from not applying the method to otoliths of all prey species.

The use of the otolith sulcus in diet studies may overcome some of the inherent challenges of species identification and fish size estimation, especially given the absence of experimental correction factors for many piscivorous predators and their prey. Studies on additional species are needed to test the applicability of the methods described here. Nevertheless, standard methods of extrapolating estimated weight from a relatively small percentage of otoliths (that is grade 0–2) averaged across predator samples in the study may misrepresent the true biomass consumed. At the least, an iterative approach to scaling otoliths Grade 0–2 would compensate for differences in prey size due to predator size (i.e., if adult predators eat larger fish than their younger counterparts) and season. Furthermore, because we cannot know if differences in estimated prey size between grade categories are only a result of digestive erosion or feeding bouts of differently sized fish (Tollit et al., 1997), perhaps using all but severely eroded otoliths provides the most representative true composition of prey size. Applying these approaches has the potential to improve the understanding of diet composition of predators and of natural mortality (consumption) of prey species in trophic studies and improve ecosystem‐based management.

## CONFLICT OF INTEREST

None declared.

## AUTHORS' CONTRIBUTIONS

BLB and AAH conceived the ideas and designed methodology; BLB, JRK, and AAH collected the data; BLB and AAH analyzed the data. All authors contributed to the writing of the manuscript and gave final approval for publication.

## Data Availability

All data used in this study are archived and available from the US Department of Commerce/National Oceanic and Atmospheric Administration at https://inport.nmfs.noaa.gov/inport/item/58439

## APPENDIX 1

**Table A1:** For otoliths from a reference collection, sample size, ∆AIC (Akaike Information Criterion) score, and r^2^ values of models used to predict fish total length (FTL, mm) and fish weight (FW, g) from otolith length (OL), sulcus length (SL), and cauda length (CL) (mm) for croaker (n = 53), spot (n = 104), spotted seatrout (n = 133), and weakfish (n = 131). Gompertz and Logistic models have 3 parameters. See Figs. A1, A2, A3, A4 for model plots

Regression	Model	Croaker		Spot		Spotted seatrout		Weakfish	
		∆AIC	r^2^	∆AIC	r^2^	∆AIC	r^2^	∆AIC	r^2^
OL‐FTL	Gompertz	0.6	0.97	0.7	0.98	1.8	0.97	0.0	0.98
	Linear	0.7	0.96	8.3	0.98	0.0	0.97	23.3	0.98
	Logistic	0.0	0.97	0.0	0.98	2.1	0.97	4.4	0.98
SL‐FTL	Gompertz	0.7	0.95	0.2	0.98	2.0	0.97	0.0	0.98
	Linear	1.6	0.95	4.1	0.98	0.0	0.97	29.6	0.97
	Logistic	0.0	0.95	0.0	0.98	2.3	0.97	3.0	0.98
CL‐FTL	Gompertz	1.6	0.88	1.1	0.95	3.2	0.97	0.0	0.97
	Linear	0.0	0.88	2.8	0.95	12.5	0.93	21.9	0.96
	Logistic	1.1	0.88	0.0	0.95	0.0	0.94	1.8	0.97
OL‐FW	Gompertz	2.3	0.96	0.0	0.97	13.7	0.95	0.0	0.93
	Linear	15.0	0.95	180.9	0.83	79.2	0.92	123.5	0.82
	Logistic	0.0	0.97	7.8	0.97	0.0	0.95	0.1	0.93
SL‐FW	Gompertz	3.4	0.94	0.0	0.97	16.1	0.94	0.4	0.93
	Linear	9.4	0.93	165.6	0.84	73.7	0.91	130.7	0.81
	Logistic	0.0	0.94	9.8	0.97	0.0	0.95	0.0	0.93
CL‐FW	Gompertz	1.9	0.89	0.0	0.93	12.6	0.89	0.0	0.92
	Linear	6.5	0.87	102.1	0.80	37.4	0.86	112.4	0.81
	Logistic	0.0	0.89	3.2	0.92	0.0	0.90	0.0	0.92
