# Supplementary material for: Using the otolith sulcus to aid in prey identification and improve estimates of prey size in diet studies of a piscivorous predator
Source: Ecol Evol. 2020 Mar 23;10(8):3584–604. doi: 10.1002/ece3.6085 (PMC7160159; doi:10.1002/ece3.6085)
Supplement: Supplementary file 1 [file ECE3-10-3584-s001.pdf]

Appendix

Table A1. For otoliths from a reference collection, sample size, ΔAIC (Akaike Information Criterion) score, and r^2 values of models used to predict fish total length (FTL, mm) and fish weight (FW, g) from otolith length (OL), sulcus length (SL), and cauda length (CL) (mm) for croaker (n = 53), spot (n = 104), spotted seatrout (n = 133), and weakfish (n = 131). Gompertz and Logistic models have 3 parameters. See Figs. A1-A4 for model plots.

		Croaker		Spot		Spotted seatrout		Weakfish	
Regression	Model	ΔAIC	r^2	ΔAIC	r^2	ΔAIC	r^2	ΔAIC	r^2
OL-FTL	Gompertz	0.6	0.97	0.7	0.98	1.8	0.97	0.0	0.98
	Linear	0.7	0.96	8.3	0.98	0.0	0.97	23.3	0.98
	Logistic	0.0	0.97	0.0	0.98	2.1	0.97	4.4	0.98
SL-FTL	Gompertz	0.7	0.95	0.2	0.98	2.0	0.97	0.0	0.98
	Linear	1.6	0.95	4.1	0.98	0.0	0.97	29.6	0.97
	Logistic	0.0	0.95	0.0	0.98	2.3	0.97	3.0	0.98
CL-FTL	Gompertz	1.6	0.88	1.1	0.95	3.2	0.97	0.0	0.97
	Linear	0.0	0.88	2.8	0.95	12.5	0.93	21.9	0.96
	Logistic	1.1	0.88	0.0	0.95	0.0	0.94	1.8	0.97
OL-FW	Gompertz	2.3	0.96	0.0	0.97	13.7	0.95	0.0	0.93
	Linear	15.0	0.95	180.9	0.83	79.2	0.92	123.5	0.82
	Logistic	0.0	0.97	7.8	0.97	0.0	0.95	0.1	0.93
SL-FW	Gompertz	3.4	0.94	0.0	0.97	16.1	0.94	0.4	0.93
	Linear	9.4	0.93	165.6	0.84	73.7	0.91	130.7	0.81
	Logistic	0.0	0.94	9.8	0.97	0.0	0.95	0.0	0.93
CL-FW	Gompertz	1.9	0.89	0.0	0.93	12.6	0.89	0.0	0.92
	Linear	6.5	0.87	102.1	0.80	37.4	0.86	112.4	0.81
	Logistic	0.0	0.89	3.2	0.92	0.0	0.90	0.0	0.92

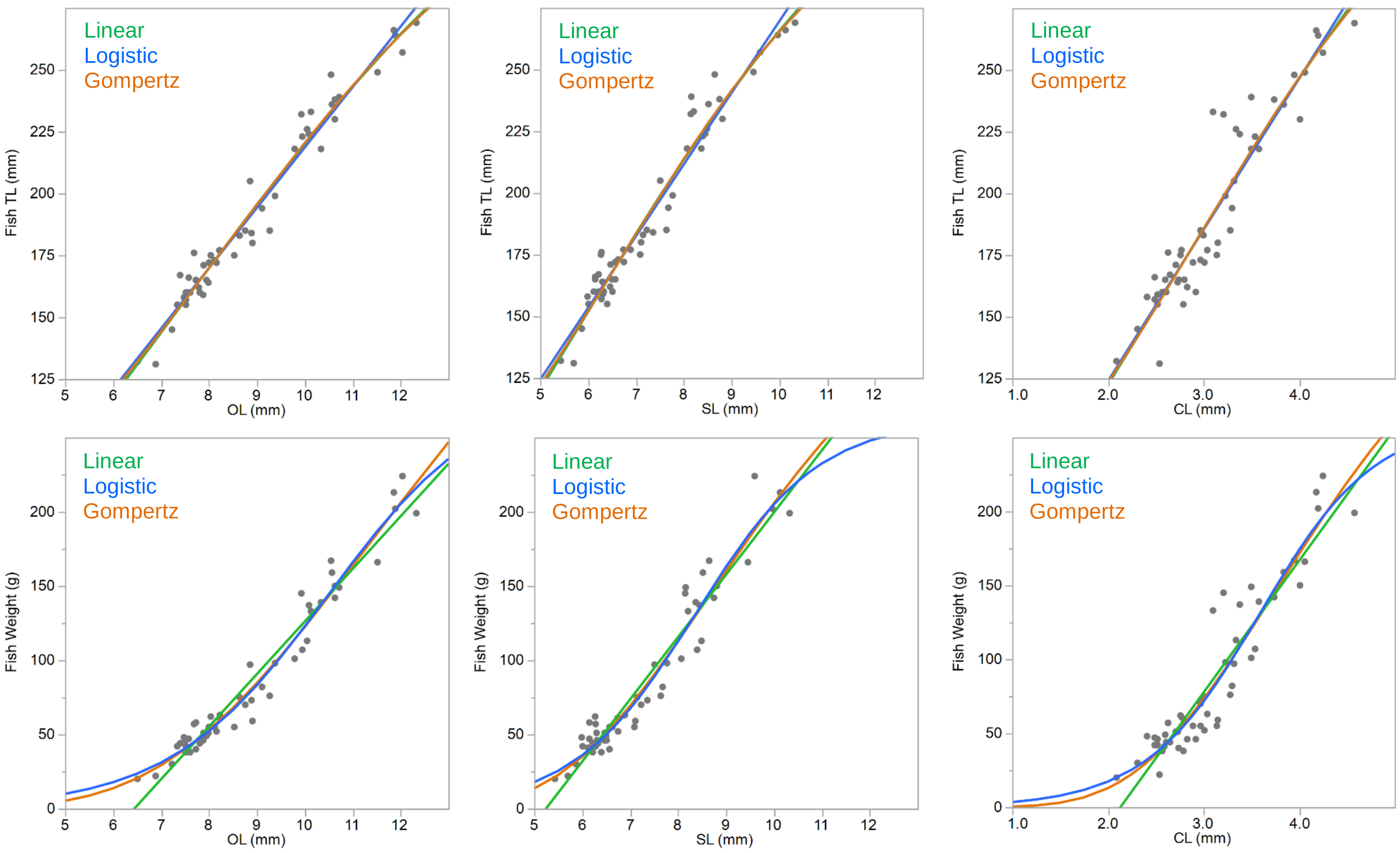

Figure A1. For croaker, Linear (green line), Logistic (blue line), and Gompertz (orange line) models were compared using ΔAkaike Information Criterion (ΔAIC) and r^2 values to determine the best fit between otolith length (OL), sulcus length (SL), and cauda length (CL), to fish total length (Fish TL, top row) and fish weight (bottom row). See Supplemental Tables A1 & A2 for ΔAIC and r^2 values.

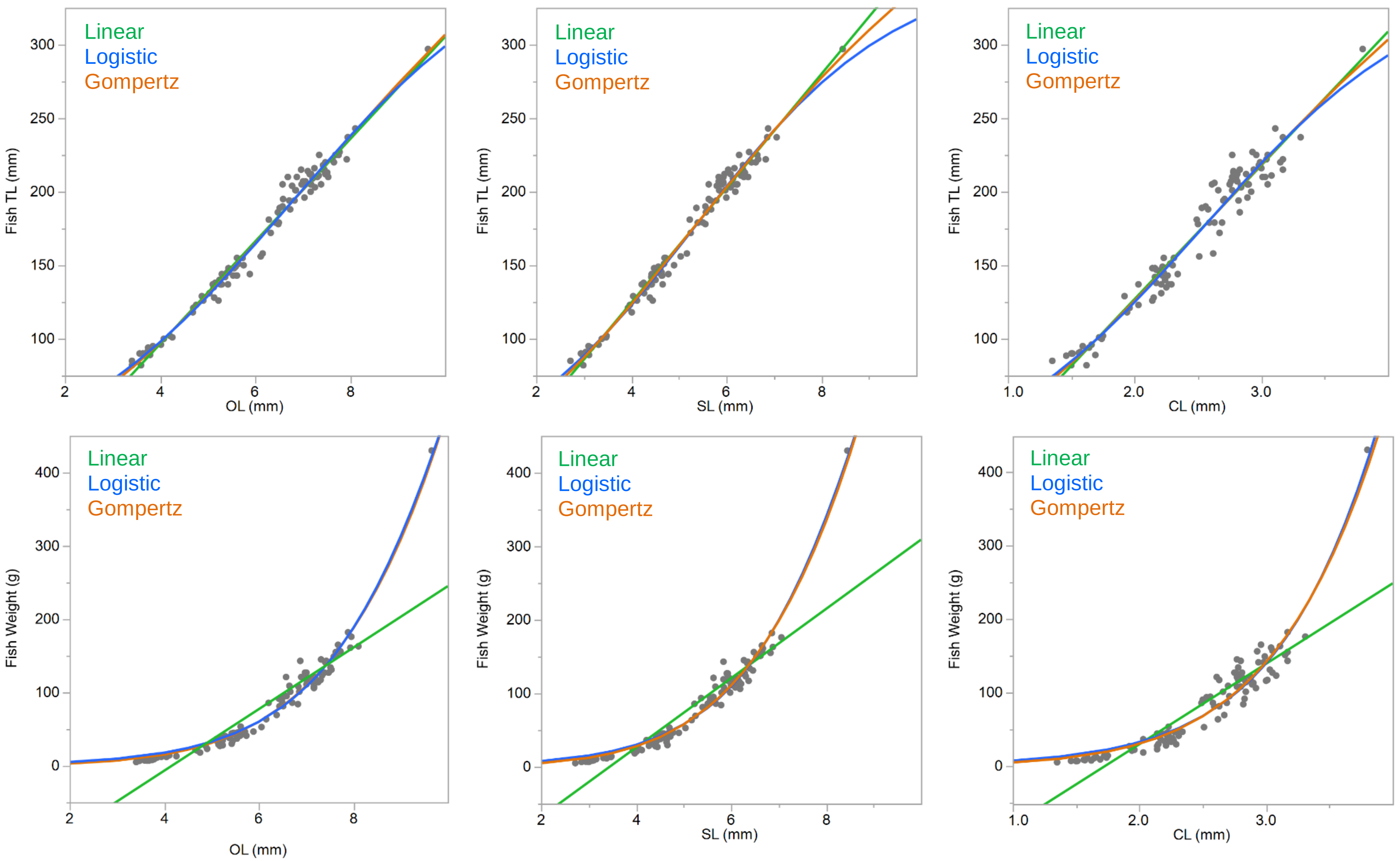

Figure A2. For spot, Linear (green line), Logistic (blue line), and Gompertz (orange line) models were compared using ΔAkaike Information Criterion (ΔAIC) and r^2 values to determine the best fit between otolith length (OL), sulcus length (SL), and cauda length (CL), to fish total length (Fish TL, top row) and fish weight (bottom row). See Supplemental Tables A1 & A2 for ΔAIC and r^2 values.

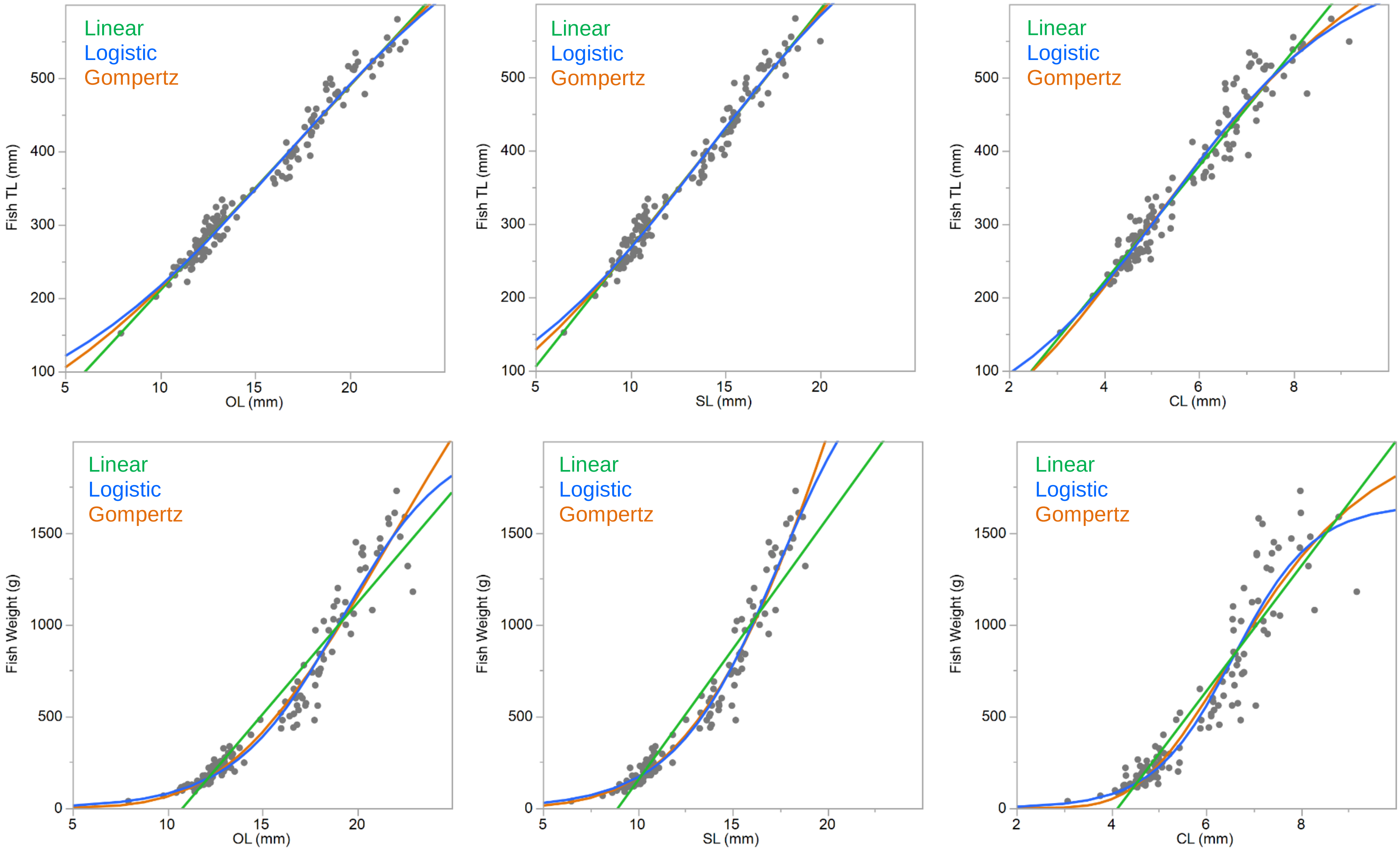

Figure A3. For spotted seatrout, Linear (green line), Logistic (blue line), and Gompertz (orange line) models were compared using ΔAkaike Information Criterion (ΔAIC) and r^2 values to determine the best fit between otolith length (OL), sulcus length (SL), and cauda length (CL), to fish total length (Fish TL, top row) and fish weight (bottom row). See Supplemental Tables A1 & A2 for ΔAIC and r^2 values.

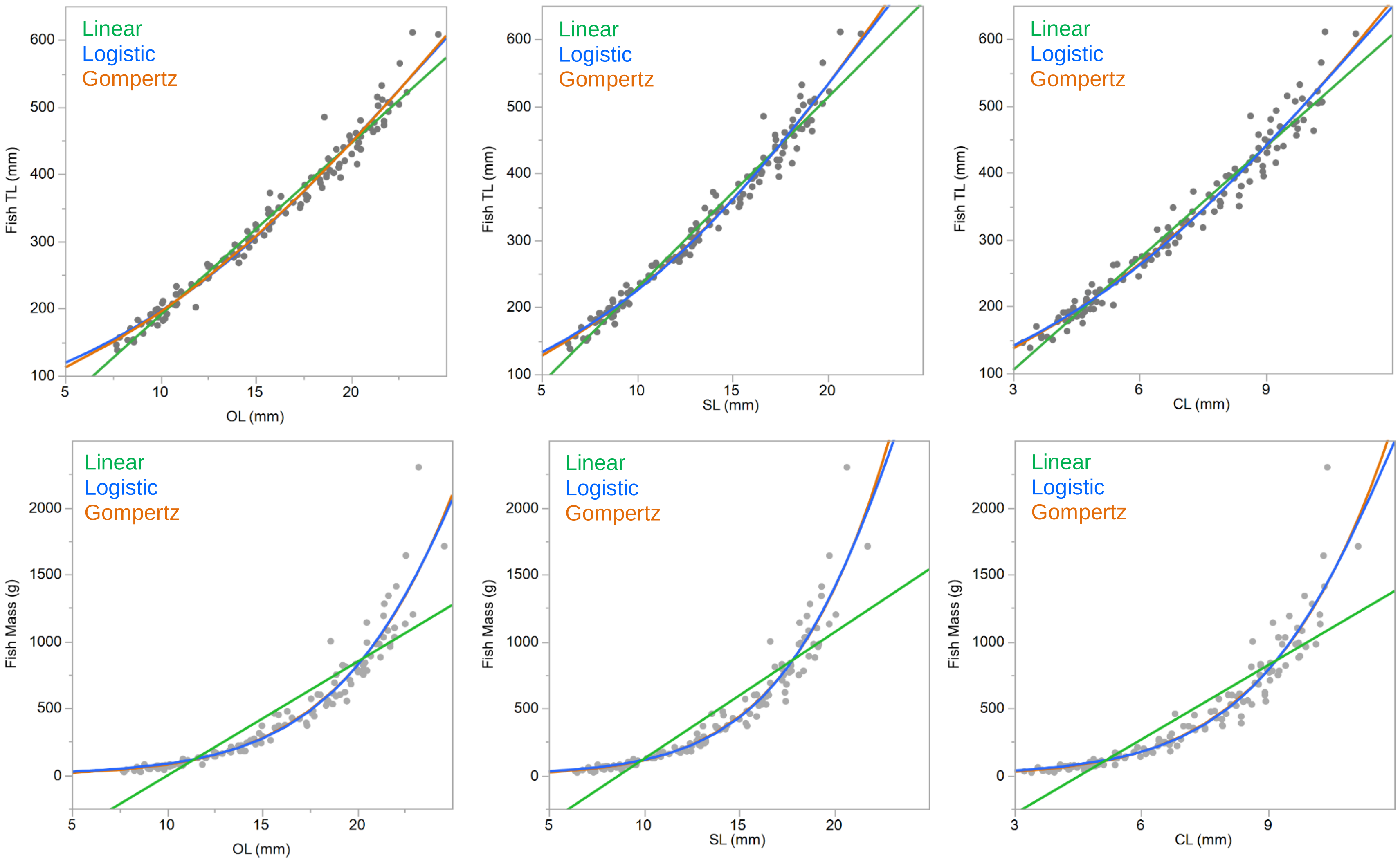

Figure A4. For weakfish, Linear (green line), Logistic (blue line), and Gompertz (orange line) models were compared using ΔAkaike Information Criterion (ΔAIC) and r^2 values to determine the best fit between otolith length (OL), sulcus length (SL), and cauda length (CL), to fish total length (Fish TL, top row) and fish weight (bottom row). See Supplemental Tables A1 & A2 for ΔAIC and r^2 values.

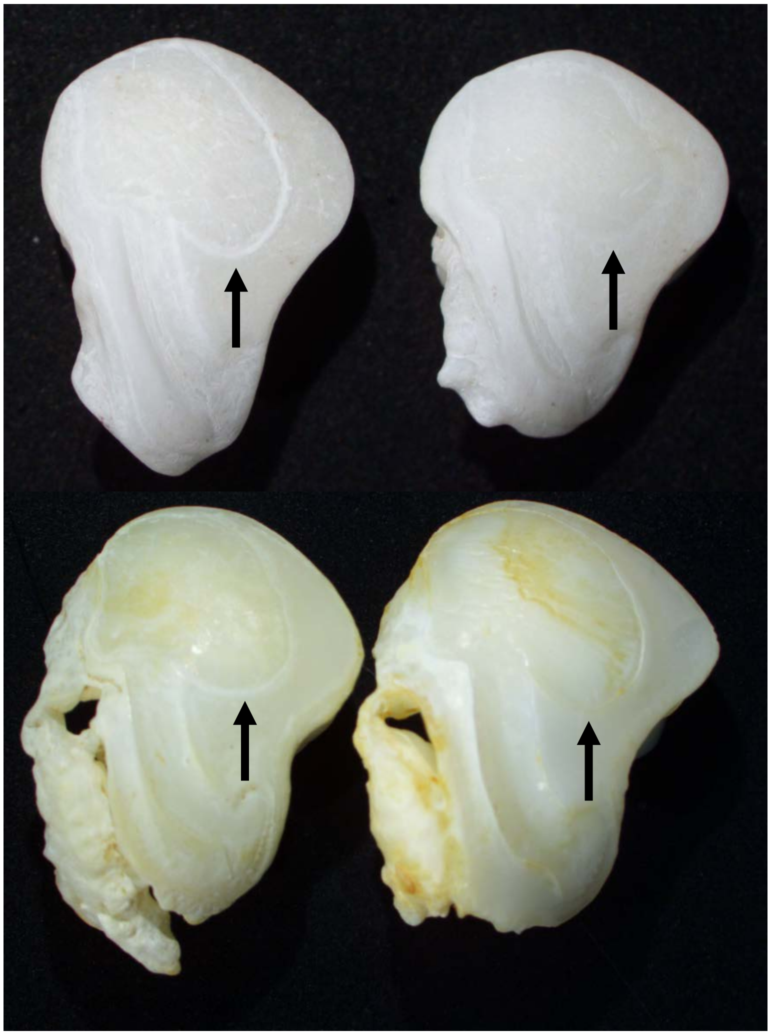

Figure A5. Croaker otoliths removed from dolphin stomachs (top: grade 2, bottom: grade 1). Notice variation in the shape and inferior position of the ostium (arrows).
